# Kinetochores Coordinate Pericentromeric Cohesion and Early DNA Replication by Cdc7-Dbf4 Kinase Recruitment

**DOI:** 10.1016/j.molcel.2013.05.011

**Published:** 2013-06-06

**Authors:** Toyoaki Natsume, Carolin A. Müller, Yuki Katou, Renata Retkute, Marek Gierliński, Hiroyuki Araki, J. Julian Blow, Katsuhiko Shirahige, Conrad A. Nieduszynski, Tomoyuki U. Tanaka

**Affiliations:** 1 Centre for Gene Regulation and Expression, College of Life Sciences, University of Dundee, Dundee DD1 5EH, UK; 2 Data Analysis Group, College of Life Sciences, University of Dundee, Dundee DD1 5EH, UK; 3 Centre for Genetics and Genomics, School of Biology, University of Nottingham, Nottingham NG7 2UH, UK; 4 Institute of Molecular and Cellular Biosciences, University of Tokyo, Tokyo 113-0032, Japan; 5 Division of Microbial Genetics, National Institute of Genetics, Mishima 411-8540, Japan; 6 Department of Genetics, School of Life Science, SOKENDAI, Mishima 411-8540, Japan

## Abstract

Centromeres play several important roles in ensuring proper chromosome segregation. Not only do they promote kinetochore assembly for microtubule attachment, but they also support robust sister chromatid cohesion at pericentromeres and facilitate replication of centromeric DNA early in S phase. However, it is still elusive how centromeres orchestrate all these functions at the same site. Here, we show that the budding yeast Dbf4-dependent kinase (DDK) accumulates at kinetochores in telophase, facilitated by the Ctf19 kinetochore complex. This promptly recruits Sld3–Sld7 replication initiator proteins to pericentromeric replication origins so that they initiate replication early in S phase. Furthermore, DDK at kinetochores independently recruits the Scc2–Scc4 cohesin loader to centromeres in G1 phase. This enhances cohesin loading and facilitates robust pericentromeric cohesion in S phase. Thus, we have found the central mechanism by which kinetochores orchestrate early S phase DNA replication and robust sister chromatid cohesion at microtubule attachment sites.

## Highlights

• The Ctf19 complex recruits Cdc7-Dbf4 kinase (DDK) to kinetochores (KTs) in telophase
• DDK at KTs recruits Sld3–Sld7 to pericentromeric origins, advancing replication timing
• DDK at KTs recruits Scc2, enhancing cohesin loading and cohesion at pericentromeres
• The DDK functions in replication timing and cohesion are independent of each other

## Graphical Abstract

**Figure:**
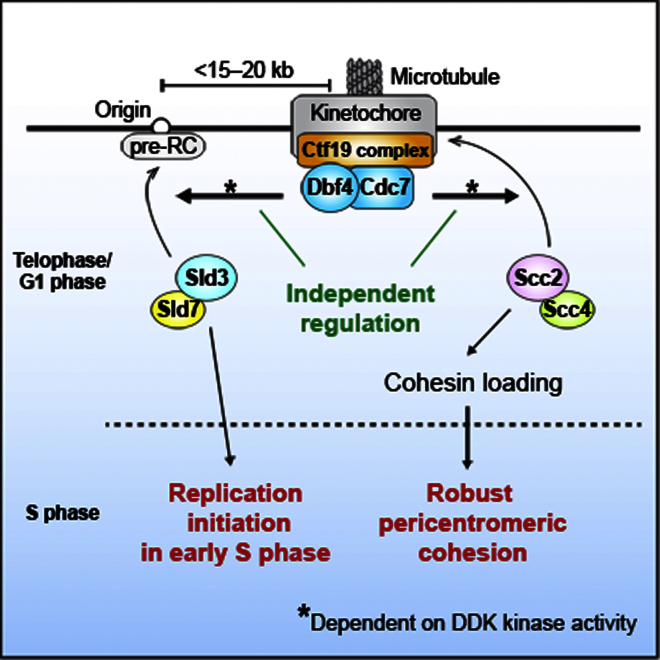

## Introduction

The centromere promotes the assembly of the kinetochore, which provides the major attachment site for spindle microtubules and ensures faithful chromosome segregation during mitosis. However, this is not the only function of the centromere; it plays at least two additional important roles. First, pericentromeres are associated with robust sister chromatid cohesion, which facilitates biorientation of sister kinetochores (Tanaka, 2002). Second, centromeric regions are replicated early during S phase in many organisms (see fourth paragraph). The early replication of centromeric DNA seems to be crucial for timely kinetochore assembly in the budding yeast *Saccharomyces cerevisiae* (Kitamura et al., 2007). Intriguingly, when a centromere is transferred to a new chromosome locus in *S. cerevisiae*, all the above features of the centromere are re-established at the new site (Hill and Bloom, 1989; Pohl et al., 2012; Tanaka et al., 1999), indicating that the centromere suffices for these functions. However, it is not known how the centromere orchestrates all these functions at the same site.

Sister chromatid cohesion relies on cohesins (Nasmyth and Haering, 2009), which are loaded onto chromatin by G1 phase, facilitated by the cohesin loader complex (called Scc2–Scc4 in *S. cerevisiae*), before the linkage between sister chromatids is established during S phase. Cohesins are enriched at pericentromeric heterochromatin by a heterochromatin protein, Swi6/HP1, in fission yeast (*Schizosaccharomyces pombe*) and human cells, which leads to the establishment of robust sister chromatid cohesion at pericentromeres (Gartenberg, 2009). In contrast, in *S. cerevisiae*, neither canonical heterochromatin nor an HP1 ortholog is present, and, instead of heterochromatin, kinetochores facilitate cohesin enrichment at pericentromeres (Tanaka et al., 1999; Weber et al., 2004). In particular, the Ctf19 kinetochore complex promotes this process by recruiting the Scc2–Scc4 complex to the centromere (Eckert et al., 2007; Fernius and Marston, 2009; Ng et al., 2009). However, it is unclear how the Ctf19 complex could enhance Scc2–Scc4 recruitment.

Meanwhile, replication of chromosomes is under temporal regulation during S phase. Each replication origin has a characteristic time window for initiating DNA replication (firing); some replication origins fire early in S phase, whereas others fire late (Natsume and Tanaka, 2010). The timing of replication initiation is set at each origin in telophase-G1 phase (Dimitrova and Gilbert, 1999; Raghuraman et al., 1997). In G1 phase of *S. cerevisiae*, some early-replicating origins, but not late-replicating ones, are loaded with Cdc7-Dbf4 (also called Dbf4-dependent kinase [DDK]), Sld3–Sld7, and Cdc45, all of which are required for replication initiation at licensed origins (i.e., origins with a prereplicative complex [pre-RC]) in S phase (Aparicio, 2013; Araki, 2011). However, it is not known how early-replicating origins are selected for loading of these factors prior to S phase.

Intriguingly, DNA replication at centromeric regions seems to be under distinct temporal regulation. For example, all 16 centromeric regions are replicated early during S phase in *S. cerevisiae* (Raghuraman et al., 2001) and other *Saccharomyces* species (Müller and Nieduszynski, 2012). Centromeric regions in other yeast species, *Candida albicans* and *S. pombe*, and in the protozoan *Trypanosoma brucei* are also replicated early in S phase (Kim et al., 2003; Koren et al., 2010; Tiengwe et al., 2012). Moreover, *Drosophila* cells show early S phase replication at core centromeres, whereas surrounding centromeric heterochromatin is replicated late in S phase (Ahmad and Henikoff, 2001). Thus, early S phase replication of centromeric regions may be a conserved feature in eukaryotic cells. However, it is unknown how the centromere facilitates early S phase replication of its DNA. Here, using *S. cerevisiae* as a model organism, we addressed these unresolved questions.

## Results

### Replication Factories Are Initially Formed at Centromeric Regions in Early S Phase

**Figure 1:**
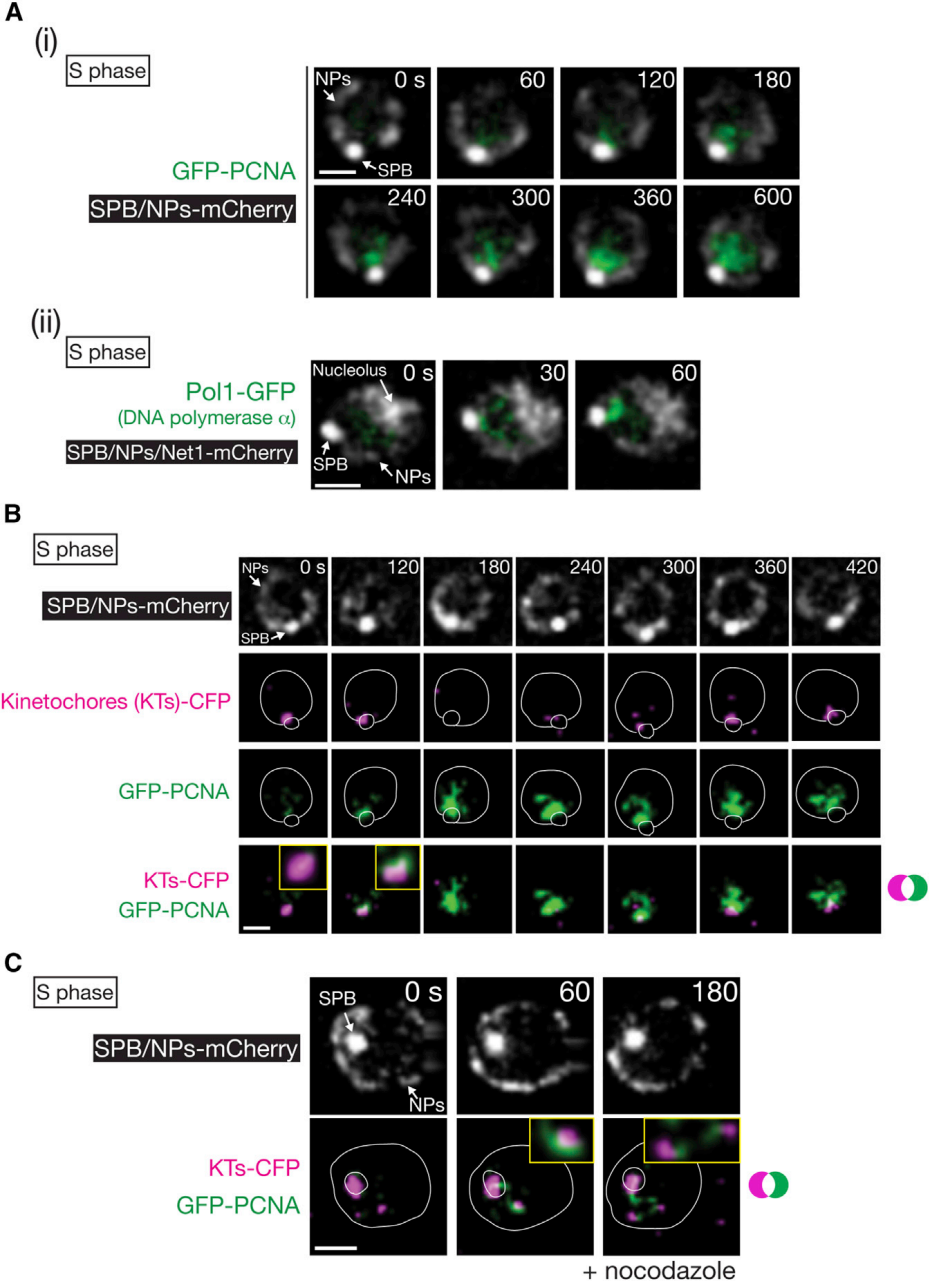
Replication Factories Are Initially Formed at Centromeric Regions (A) Replication factories are initially formed near the SPB. *GFP-PCNA* (T8375 [i]) and *POL1*-*GFP* (T7300 [ii]) cells with *SPC42-mCherry NIC96-mCherry* (visualizing SPBs and nuclear pores [NPs]) were observed in asynchronous culture. T7300 cells also had *NET1-mCherry* (nucleolus marker). We set 0 s arbitrarily. The scale bar represents 1 μm. (B) Replication factories are initially formed, overlapping with the kinetochore cluster. Cells (T10459) with *GFP-PCNA SPC42-mCherry NIC96-mCherry NDC80-CFP* (visualizing kinetochores [KTs]) were observed in asynchronous culture. White lines represent the nucleus and the SPB. CFP, cyan fluorescent protein. (C) Replication factories are initially formed on centromeres when they are detached from a spindle pole. Cells (T8819) with *GFP-PCNA MTW1-CFP NDC80-CFP* (KTs-CFP) *SPC42-mCherry NIC96-mCherry mad2*Δ *bub2*Δ were arrested with α factor and released into medium with nocodazole. Kinetochores were frequently detached from a spindle pole after going through S phase in the presence of nocodazole. *mad2*Δ *bub2*Δ allowed cells to exit from mitosis in the absence of microtubules. Cells were observed after cytokinesis (125 min after release from α factor).

In budding yeast, as in animals, the majority of DNA replication occurs in replication factories, which contain DNA polymerases and other replication proteins (Natsume and Tanaka, 2010). To observe replication factories, we visualized proliferating cell nuclear antigen (PCNA), a sliding clamp for DNA polymerases. Live-cell imaging showed that replication factories initially formed adjacent to the spindle pole body (SPB, equivalent to the centrosome in metazoa) (Figure 1Ai, at 60 and 120 s) and subsequently spread into the whole nucleus (at 600 s). A similar result was obtained by visualizing Pol1, a catalytic subunit of DNA polymerase α (Figure 1Aii).

In budding yeast, all kinetochores (formed at centromeres) are tethered to the SPB by microtubules and clustered adjacent to the SPB (Duan et al., 2010; Guacci et al., 1997), raising the possibility that replication factories are initially formed at centromeric regions. Indeed, replication factories, when initially formed, overlapped with the cluster of kinetochores (in all 14 cells observed) (Figure 1B, at 120 s). Because of kinetochore clustering near the SPB, the density of chromosomes is high in the vicinity of the SPB. However, this was not the sole reason for the initial emergence of replication factories there; in contrast, histone H2B and Orc2, a component of the pre-RC at replication origins, did not show such a clear accumulation at this region (data not shown).

To address whether replication factories are initially formed at centromeric regions, we detached centromeres from the SPB prior to S phase by treating cells with nocodazole, which depolymerized microtubules (Figure 1C). In such conditions, replication factories were initially observed at detached kinetochores that were distant from the SPB, as well as in the vicinity of kinetochores that remained near the SPB. This indeed suggests that replication factories are initially formed at centromeric regions. It is known that replication factories are assemblies of replication forks and are formed as a consequence of DNA replication (Kitamura et al., 2006). Therefore, it is probable that the initiation of factory formation at centromeric regions reflects their early S phase replication. Indeed, soon after the appearance of factories at clustered kinetochores, the kinetochores transiently reduced their signal intensity (Figure 1B, 180–300 s), consistent with the kinetochore disassembly and centromere detachment from microtubules, caused by replication of centromeres (Kitamura et al., 2007).

### Cdc7-Dbf4 and Sld3–Sld7 Localize at Centromeric Regions in Telophase to G1 Phase

**Figure 2:**
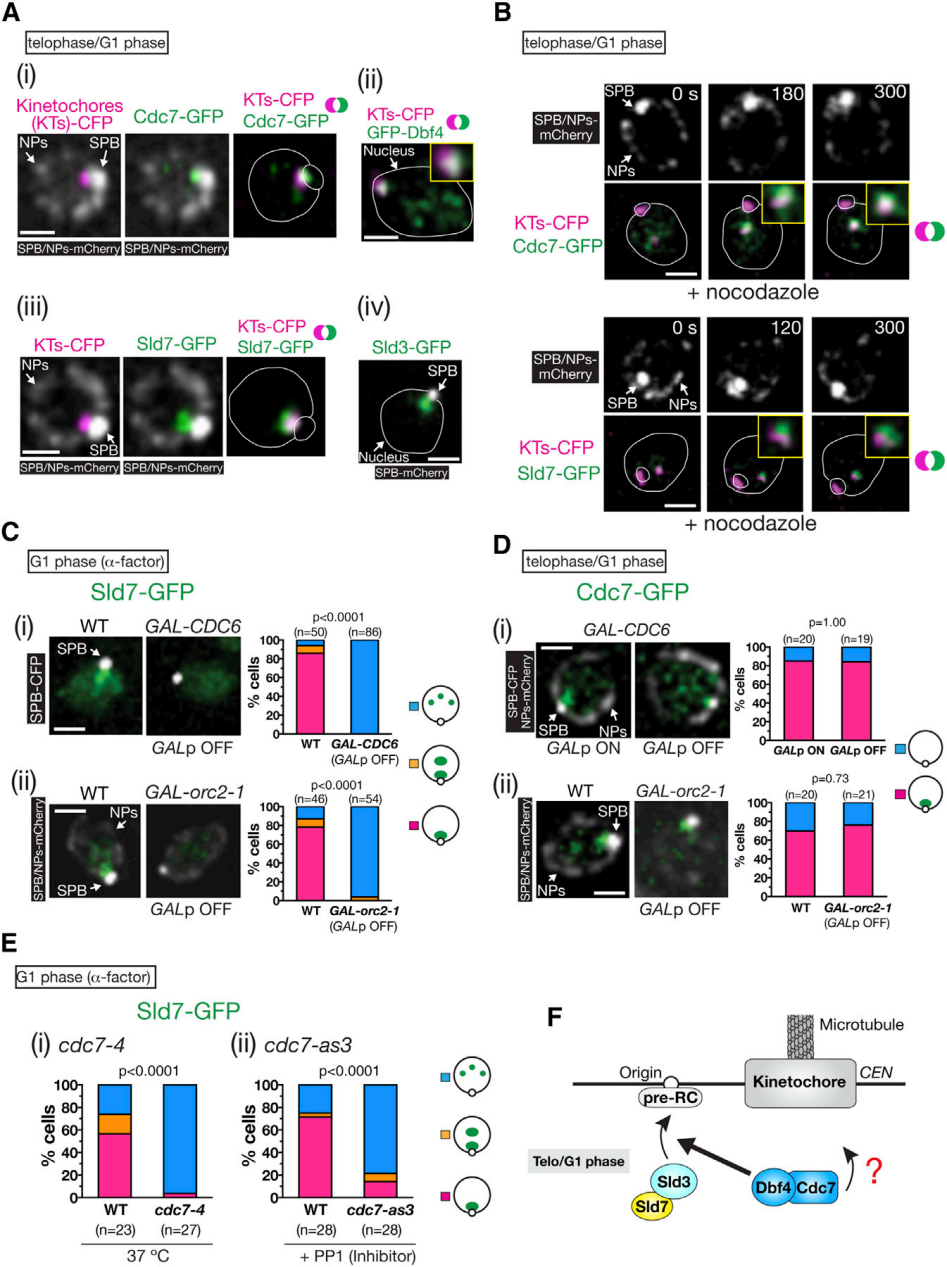
DDK and Sld3–Sld7 Localize at Centromeric Regions from Telophase to G1 Phase (A) DDK and Sld3–Sld7 localize at centromeric regions from telophase to G1 phase. Cells with *CDC7-GFP MTW1-CFP NDC80-CFP SPC42-mCherry NIC96-mCherry* (T10876 [i]), *GFP-DBF4 MTW1-CFP NDC80-CFP* (T9466 [ii]), *SLD7-GFP NDC80-CFP SPC42-mCherry NIC96-mCherry* (T10875 [iii]), and *SLD3-GFP SPC42-mCherry* (T8784 [iv]) were observed in asynchronous culture. Cells in telophase–G1 phase were selected. The scale bar represents 1 μm. (B) Cdc7 and Sld7 colocalize with centromeres when they are detached from a spindle pole. T8888 and T8886 cells (same as T10876 and T10875 but with *mad2*Δ *bub2*Δ) were observed as in Figure 1C. (C) Sld7 localization at centromeric regions is dependent on pre-RC. *CDC6*^*+*^ (wild-type [WT], T9126) and *GAL-CDC6* (T9127) cells with *SLD7-GFP SPC42-CFP* (i), as well as *ORC2*^*+*^ (WT, T9273) and *GAL-orc2-1* (T9272) cells with *SLD7-GFP SPC42-mCherry NIC96-mCherry rad9*Δ *rad24*Δ *mad2*Δ (ii), were treated with α factor, released from it, and treated with α factor again. *GAL* promoter (*GALp*) was shut off during this process (see Supplemental Experimental Procedures). Cells were observed after cytokinesis. (D) Cdc7 localization on centromeric regions is independent of pre-RC. *GAL-CDC6* cells (T9125) with *CDC7-GFP SPC42-CFP NIC96-mCherry* (i), as well as *ORC2*^*+*^ (WT, T9275) and *GAL-orc2-1* (T9274) cells with *CDC7-GFP SPC42-mCherry NIC96-mCherry rad9*Δ *rad24*Δ *mad2*Δ (ii), were treated with α factor, released from it, and observed in 120 min. *GALp* was active (ON) or shut off (OFF). (E) Cdc7 and its kinase activity are required for Sld7 localization on centromeric regions. (i) *CDC7*^*+*^ (WT, T8613) and *cdc7-4* (T9085) cells with *SLD7-GFP SPC42-mCherry* were incubated at 25°C for 2.5 hr and then at 37°C for 35 min, in the presence of α factor. (ii) *CDC7*^*+*^ (WT, T8613) and *cdc7-as3* (T10417) cells with *SLD7-GFP SPC42-mCherry* were arrested in G1 phase with α factor and then treated with 20 μM PP1 (inhibitor) for 20 min in the presence of α factor. (F) Summary of results in Figure 2. See details in the text. See also Figure S1.

What mechanism promotes the initiation of replication-factory formation at centromeric regions? Replication initiation proteins may preferentially localize to these regions prior to S phase. In particular, their localization in telophase–early G1 phase may be important because DNA replication timing is set during this stage of the cell cycle (Dimitrova and Gilbert, 1999; Raghuraman et al., 1997). In live-cell imaging, cells in telophase–early G1 phase were identified by full segregation of SPBs (due to full spindle elongation) and their subsequent repositioning following spindle disassembly (Figure S1A available online). We investigated the subnuclear localization of a range of replication initiation proteins in telophase–early G1 phase. Cdc45 and Sld2 did not show accumulation near the SPB, whereas Clb5 (an S phase cyclin) and Cdc28 (Cdk1) localized there only from late S phase to metaphase (data not shown). By contrast, Dbf4, Cdc7, Sld3, and Sld7 showed clear accumulation near the SPB and close localization to kinetochores at telophase–early G1 phase (Figure 2A). Moreover, they also localized in the vicinity of kinetochores that had been moved away from the SPB after nocodazole treatment (Figure 2B). Thus, DDK and the Sld3–Sld7 complex are candidate regulators for initial factory formation at centromeric regions.

We next addressed how DDK and Sld3–Sld7 localize at centromeric regions. Both factors can be loaded on replication origins at which the pre-RC is assembled (Araki, 2011; Labib, 2010). We therefore investigated whether the accumulation of DDK and Sld3–Sld7 at centromeric regions is dependent on pre-RC components Cdc6 and Orc2. After depletion of either Cdc6 or Orc2, the localization of Sld7 to centromeric regions in G1-arrested cells was abolished (Figure 2C), but Cdc7 remained localized there (Figure 2D). These results suggest that Sld3–Sld7 associates with replication origins at pericentromeres, whereas DDK localizes at centromeric regions in a pre-RC-independent manner, and presumably in an origin-independent manner.

Next, we investigated the temporal order of localization of DDK and Sld3–Sld7 at centromeric regions. Cdc7 accumulated near the SPBs in telophase–early G1 phase, followed by Sld7 accumulation there (Figure S1B). Whereas the Cdc7 signal subsequently faded, Sld7 accumulation continued until late G1 phase. Subsequent disappearance of Sld7 near the SPB was almost coincidental with emergence of replication factories in the region (Figure S1C). To test whether DDK is required for the Sld3–Sld7 localization at centromeric regions, we used a temperature-sensitive mutant, *cdc7-4*, and an ATP-analog-sensitive mutant, *cdc7-as3* (Figure 2E). Inactivation of Cdc7 kinase reduced Sld7 localization at centromeric regions in G1-arrested cells, suggesting that DDK activity is required for the association of Sld3–Sld7 with replication origins at pericentromeres in telophase to G1 phase (Figure 2F).

### A Kinetochore Component, the Ctf19 Complex, Recruits DDK and Facilitates Replication of Centromeric Regions in Early S Phase

DDK recruitment to centromeric regions may facilitate the loading of Sld3–Sld7 on pericentromeric replication origins, leading to their early S phase replication initiation (early firing). To test this, we aimed to identify the mechanism for DDK recruitment to centromeric regions. We investigated Sld7 accumulation near the SPB in mutants of a number of candidate regulators, which are associated with centromeres and kinetochores (Figure S2A). We visualized Sld7, rather than DDK, because the intensity of DDK near the SPB was highly variable among cells, making its quantitative evaluation difficult.

**Figure 3:**
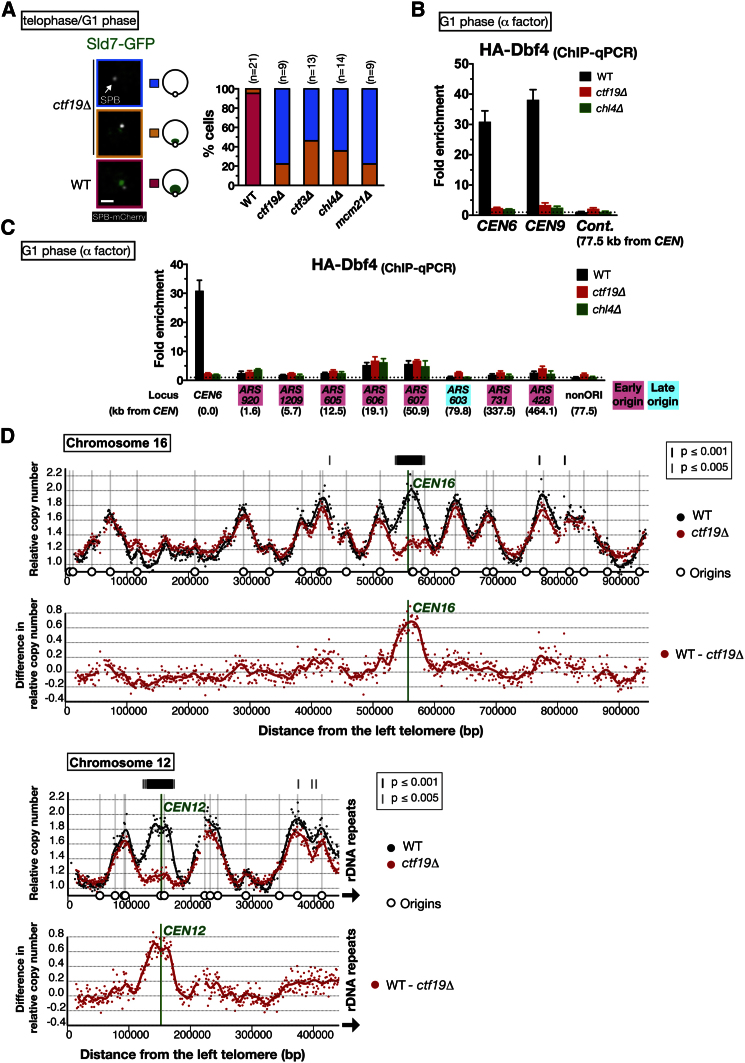
The Ctf19 Complex Recruits DDK to Centromeres and Facilitates Early Replication of Centromeric Regions (A) Sld7 localization on centromeric regions is abolished in mutants of the Ctf19-complex components. WT (T8613), *ctf19*Δ (T9650), *ctf3*Δ (T9708), *chl4*Δ (T9709), and *mcm21*Δ (T9710) cells with *SLD7-GFP SPC42-mCherry* were observed in asynchronous culture. Cells in telophase–G1 phase were selected. The scale bar represents 1 μm. (B) Dbf4 association with centromeres is reduced in *ctf19*Δ and *chl4*Δ cells. WT (T9945), *ctf19*Δ (T10275), and *chl4*Δ (T10278) cells with *HA-DBF4* were treated with α factor for 2.5 hr and processed for ChIP using a hemagglutinin (HA) antibody. Coprecipitated DNA was analyzed with qPCR at *CEN6*, *CEN9*, and a control locus (*PHO4*, 77.5 kb from *CEN6*). The ratio of immunoprecipitated DNA to total DNA in whole-cell extract is normalized relative to a control locus in WT (fold enrichment). Error bars represent SD. (C) Dbf4 association with replication origins in G1 phase. ChIP-qPCR was performed as in (B) and analyzed at replication origins and the nonorigin (nonORI) locus (*PHO4*). Error bars represent SD. (D) Replication of centromeric regions is specifically delayed in *ctf19*Δ cells. S phase and G2–M phase cells were collected from a culture of WT (T9475) and *ctf19*Δ (T10117) homozygous diploids. The ratio of the copy number in S phase cells to that in G2–M phase cells is normalized and shown between 1.0 and 2.0 at each chromosome locus. The difference in the replication timing between the two strains is shown at bottom. Smoothed lines were added in both graphs. See also Figures S2 and S3.

The Ctf19 complex (also called COMA) is a conserved kinetochore component that links inner kinetochore components to outer ones (Schleiffer et al., 2012 and references therein). Deletion of some components of this complex can still yield viable yeast cells. We found that deletion of *CTF19*, encoding a component of the Ctf19 complex, reduced Sld7 localization near the SPB in telophase to G1 phase (Figure 3A). Nonetheless, in most *ctf19*Δ cells, *CEN2* stayed in the vicinity of SPB during these phases (Figure S2B), suggesting that loss of Sld7 accumulation was not due to a lack of centromere clustering near the SPB. The deletion of other components of the Ctf19 complex, *CTF3*, *CHL4*, and *MCM21*, showed similar results (Figure 3A). In contrast, the deletion of other nonessential kinetochore components such as *CNN1* and *SLK19* showed normal Sld7 accumulation near the SPB (Figure S2A), suggesting that the Ctf19 complex has a distinct role in this process on kinetochores.

Next, we examined DDK localization at centromeric regions in G1-arrested cells, using a chromatin immunoprecipitation assay in which coimmunoprecipitated DNA was analyzed with quantitative PCR (ChIP-qPCR). Dbf4 association with centromeres was detected in wild-type cells arrested in G1 phase (Figure 3B), consistent with our microscopy result (see Figure 2Aii). In *ctf19*Δ and *chl4*Δ cells, however, this association was reduced considerably. Thus, the Ctf19 complex promotes DDK association with centromeres in telophase–G1 phase. Meanwhile, DDK is also loaded on early-firing origins in G1 cells (Katou et al., 2006). However, we found that Dbf4 association with early-firing origins (such as *ARS606* and *ARS607*) did not change in *ctf19*Δ or *chl4*Δ cells (Figure 3C). Therefore, the Ctf19 complex specifically regulates the kinetochore-associated DDK, but not the origin-associated DDK.

Furthermore, deletion of *CTF19* also led to reduction of the initial formation of replication factories at centromeric regions (Figure S2C). Thus, early S phase centromere replication may be perturbed in *ctf19*Δ cells. To test this, we compared the whole-genome replication-timing profile between wild-type and *ctf19*Δ cells (Figures 3D and S3). For this, S phase and G2–M phase cells were fractionated by cell sorting on the basis of DNA content. Then, using high-throughput DNA sequencing, the number of DNA sequence reads in S phase cells was quantified relative to that in G2–M phase cells. The earlier a sequence is replicated in S phase, the more reads of the sequence are obtained in the S phase sample (Müller and Nieduszynski, 2012). In both wild-type and *ctf19*Δ cells, the peaks of replication-timing profiles coincided with positions of replication origins. In wild-type cells, all centromeric regions showed early replication in S phase (Figures 3D and S3), consistent with previous reports (Raghuraman et al., 2001). Wild-type and *ctf19*Δ cells showed similar replication profiles along chromosome arms. Remarkably, replication of centromeric regions (up to 50 kb from centromeres) was specifically delayed in *ctf19*Δ cells. The extent of this delay varied from chromosome to chromosome (Figure S3). The maximum delay was observed on chromosomes 9, 12, and 16. In centromeric regions where replication was delayed in *ctf19*Δ cells, we still detected small peaks at replication origins, suggesting that these origins were still active but that replication initiation was delayed. Thus, the Ctf19 complex facilitates early S phase replication of centromeric regions.

### DDK Recruitment to Kinetochores Facilitates Replication of Centromeric Regions in Early S Phase

**Figure 4:**
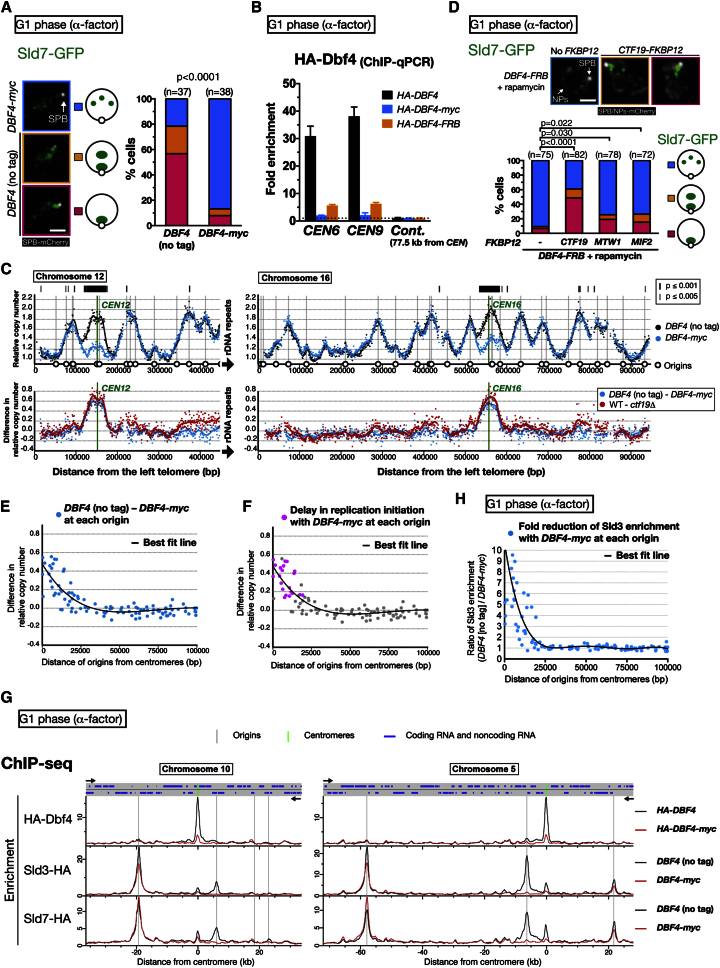
DDK at Kinetochores Advances Replication of Centromeric Regions in S Phase (A) Sld7 localization is abolished at centromeric regions in G1 phase when Dbf4 is tagged at its C terminus. *DBF4* (no tag) (T8613) and *DBF4-myc* (T9396) cells with *SLD7-GFP SPC42-mCherry* were treated with α factor for 2.5 hr. The scale bar represents 1 μm. (B) Dbf4 association with centromeres is reduced in G1 phase when Dbf4 is tagged with Myc or FRB at its C terminus. *HA-DBF4* (T9945), *HA-DBF4-myc* (T9979), and *HA-DBF4-FRB* (T10360) cells were analyzed as in Figure 3B. Error bars represent SD. (C) Replication of centromeric regions is specifically delayed in *DBF4-myc* cells similarly to *ctf19*Δ cells. *DBF4* (no tag) (T9475) and *DBF4-myc* (T9476) homozygous diploid cells were analyzed as in Figure 3D. Replication timing (top) and its difference (bottom) are shown. (D) Artificial tethering of Dbf4-FRB to Ctf19-FKBP12 restores Sld7 localization on centromeric regions in G1 phase. *DBF4-FRB SLD7-GFP SPC42-mCherry NIC96-mCherry* cells with either *CTF19-FKBP12* (T10149), *MTW1-FKBP12* (T10147), *MIF2-FKBP*12 (T10148), or no *FKBP12* tag (T9915), were treated with α factor for 2.5 hr in the presence of 10 μM rapamycin. (E) Replication timing of origins in *DBF4* (no tag) and *DBF4-myc* cells. Difference in replication timing between in *DBF4* (no tag) and *DBF4-myc* cells (see C) is plotted for origins against the distance from the centromere. A regression curve is shown as a black line. (F) Pericentromeric origins up to 15–20 kb from centromeres show delays in their replication initiation in *DBF4-myc* cells. Replication origins showing the delay in replication initiation, obtained with mathematical modeling, are marked in magenta in the graph presented in (E). (G) ChIP-seq analysis of Dbf4 and Sld3–Sld7 localization in G1 phase. *HA-DBF4* (T9945), *HA-DBF4-myc* (T9979), *SLD3-HA DBF4* (no tag) (T9861), *SLD3-HA DBF4-myc* (T9970), *SLD7-HA DBF4* (no tag) (T9862), and *SLD7-HA DBF4-myc* (T9971) cells were treated with α factor for 2.5 hr and analyzed via ChIP-seq (immunoprecipitation [IP] with an HA antibody). (H) Pericentromeric origins up to 15–20 kb from centromeres show reduction in their association with Sld3 in *DBF4-myc* cells. The ratio of Sld3 enrichment (*DBF4* [no tag]/*DBF4-myc*), obtained with ChIP-seq (Figure 4G), is plotted for origins against the distance from the centromere. A regression curve is shown as a black line. See also Figures S3 and S4.

The above results raised the possibility that DDK at kinetochores is responsible for advancing pericentromere replication timing. We can test this possibility if we find a *dbf4* mutant in which DDK localization at kinetochores is defective in telophase–early G1 phase but S phase progression is still supported normally. Fortuitously, *DBF4* tagged at its C terminus demonstrated these properties, as follows: First, in cells expressing *DBF4-myc* (tagged at the C terminus), the appearance of Sld7 near the SPB became rare in G1 phase (Figure 4A), whereas it was frequent with *myc-DBF4* (tagged at the N terminus), similar to untagged *DBF4* (Figure S4A). Second, with C-terminally tagged *DBF4-myc*, formation of replication factories became infrequent near the SPB, in contrast to untagged *DBF4* (Figure S4B). Third, using ChIP-qPCR and ChIP followed by high-throughput DNA sequencing (ChIP-seq), we found that C-terminal tagging (*myc* or *FRB*) of *DBF4* caused a dramatic reduction of Dbf4 bound at centromeres (Figure 4B; see Figure 4G), but not at replication origins (Figures S4C and S4D). Fourth, although C-terminal tagging of Dbf4 impaired DDK localization at kinetochores in telophase–G1 phase, S phase progression was largely normal with *DBF4-myc* when DNA contents were measured by fluorescence-activated cell sorting (FACS) (Figure S4E). Fifth, *DBF4-myc* appears to function like untagged *DBF4* at replication origins on chromosome arms because, although Sld3 recruitment to replication origins requires DDK (Heller et al., 2011; Tanaka et al., 2011), this occurred normally with *DBF4-myc*, except for in centromeric regions (see Figure 4H).

How would a DNA replication profile be changed if the level of DDK were specifically reduced at kinetochores? Crucially, *DBF4-myc* cells showed a delay in replication timing at centromeric regions specifically, and their replication profile was very similar to that of *ctf19*Δ cells (Figures 4C and S3). Therefore, the Ctf19 complex recruits DDK to kinetochores in telophase–early G1 phase, and this DDK at kinetochores advances replication timing of centromeric regions. This notion is further supported by restoration of Sld7 accumulation and replication-factory initiation near the SPB when Dbf4-FRB was tethered to Ctf19-FKBP12 (Figures 4D and S4F), using rapamycin-dependent association between FRB and FKBP12 (Chen et al., 1995). By contrast, this restoration was less effective when Dbf4 was tethered to other kinetochore components such as Mtw1 and Mif2 (Figure 4D).

The essential function of DDK in replication is to recruit several replication proteins to replication origins (autonomously replicating sequences [ARSs]) and to promote replication initiation there (Labib, 2010). DDK loaded at kinetochores may advance replication timing of centromeric regions by recruiting the Sld3–Sld7 complex (see Figure 2F) and other replication initiation proteins to pericentromeric origins in telophase–early G1 phase. If so, how far does this effect reach to pericentromeric origins? In *DBF4-myc* and *ctf19*Δ cells, the replication delay was greatest in the close proximity of centromeres but became smaller when further away from centromeres (Figures 4E and S4G). Nonetheless, the delay was found up to 20–25 kb away. Such a delay might be due to a delayed replication initiation at a relevant origin, but might also be due to a delay in replication initiated from other origins. We used mathematical modeling to distinguish these two effects (de Moura et al., 2010) and recapitulated the replication-timing profile of *DBF4-myc* cells by delaying replication initiation of pericentromeric origins up to 15–20 kb from centromeres (Figure 4F). Furthermore, consistent with this result, using ChIP-seq we found that the amount of Sld3 and Sld7, bound to replication origins, was reduced up to 15–20 kb from centromeres in *DBF4-myc* cells (Figures 4G, 4H, and S4H). Thus, DDK at kinetochores recruits Sld3–Sld7 to pericentromeric origins up to 15–20 kb from centromeres and advances timing of their replication initiation.

This explains why delays in replication timing found at centromeric regions of *DBF4-myc* and *ctf19*Δ cells are larger on some chromosomes but smaller on others (Figure S3). In these cells, the origins up to 15–20 kb from centromeres would show delays in replication initiation. However, if the next early-firing origin is located just outside this region, replication from this origin (whose initiation timing is not delayed) alleviates the replication delay (Figure S4I). By contrast, if the next origin is far away, a larger delay occurs along a wider region.

### DDK Recruited to Kinetochores Facilitates Robust Sister Chromatid Cohesion at Pericentromeres

**Figure 5:**
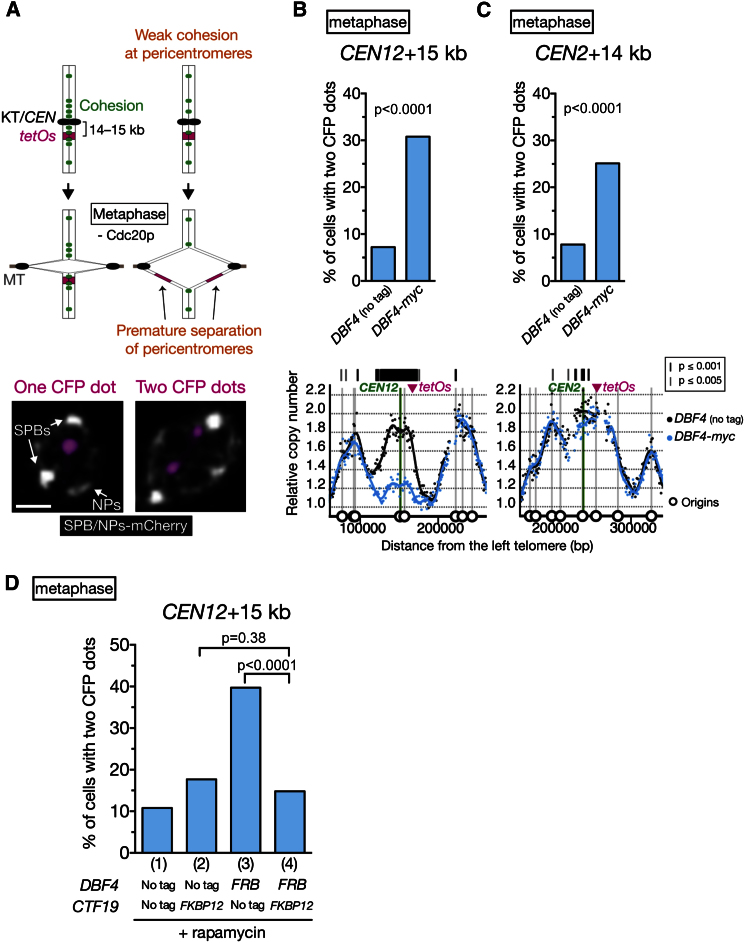
Kinetochore-Associated DDK Facilitates Robust Sister Chromatid Cohesion at Pericentromeres (A) An assay for evaluating cohesion at pericentromeres. The *tetOs*, integrated at 14–15 kb from a centromere, were bound with TetR-CFP. Cells with *CDC20* under *MET3* promoter were treated with α factor, released to methionine-containing medium (for depletion of Cdc20), and arrested in metaphase. At 2 hr after the release, the percentage of cells with two sister CFP dots was counted. The scale bar represents 1 μm. (B and C) Sister chromatid cohesion is weakened at the *CEN12* and *CEN2* pericentromeres in *DBF4-myc* cells. *DBF4* (no tag) (T10141, n = 249) and *DBF4-myc* (T10142, n = 266) cells with *tetOs* at +15 kb from *CEN12* (B), or those (T10194, n = 347; T10195, n = 299; respectively) with *tetOs* at +14 kb from *CEN2* (C), were treated and analyzed as in (A). The replication-timing profile of the pericentromeres is shown at the bottom. (D) Artificial tethering of Dbf4-FRB to Ctf19-FKBP12 restores pericentromeric cohesion. Cells with indicated alleles of *DBF4* and *CTF19*, as well as *tetOs* at +15 kb from *CEN12*, were treated and analyzed as in (A). During α factor treatment and thereafter, 10 μM rapamycin was added. n = 305–353 in each condition. See also Figure S5.

It has been reported that the Ctf19 complex enhances cohesin recruitment to pericentromeres, which facilitates robust sister chromatid cohesion there (Eckert et al., 2007; Fernius and Marston, 2009; Ng et al., 2009). However, it remains unclear what factors mediate this process. Given that the Ctf19 complex promotes DDK recruitment to kinetochores, DDK recruited to kinetochores might be a facilitator of robust pericentromeric cohesion. We therefore evaluated pericentromeric cohesion at pericentromeres during metaphase. The pericentromeric loci showed more frequent sister chromatid separation in *DBF4-myc* cells compared with control cells (i.e., untagged *DBF4*) (Figures 5A–5C). This is consistent with pericentromeric cohesion being weakened in *DBF4-myc* cells. Then, is the weakened pericentromeric cohesion indeed due to a lack of DDK at kinetochores? We tested this by restoring kinetochore localization of Dbf4-FRB, which in itself showed considerable reduction at kinetochores, similarly to Dbf4-myc (see Figure 4B). After Dbf4-FRB was tethered to kinetochores in the presence of Ctf19-FKBP12 and rapamycin, separation of sister chromatids was alleviated at pericentromeres during metaphase (Figure 5D). Thus, we suggest that DDK recruitment to kinetochores, promoted by the Ctf19 complex, facilitates robust sister chromatid cohesion at pericentromeres. This notion is also supported by the evaluation of pericentromeric cohesion in the combination of *DBF4-myc* with *chl4*Δ (Figure S5).

**Figure 6:**
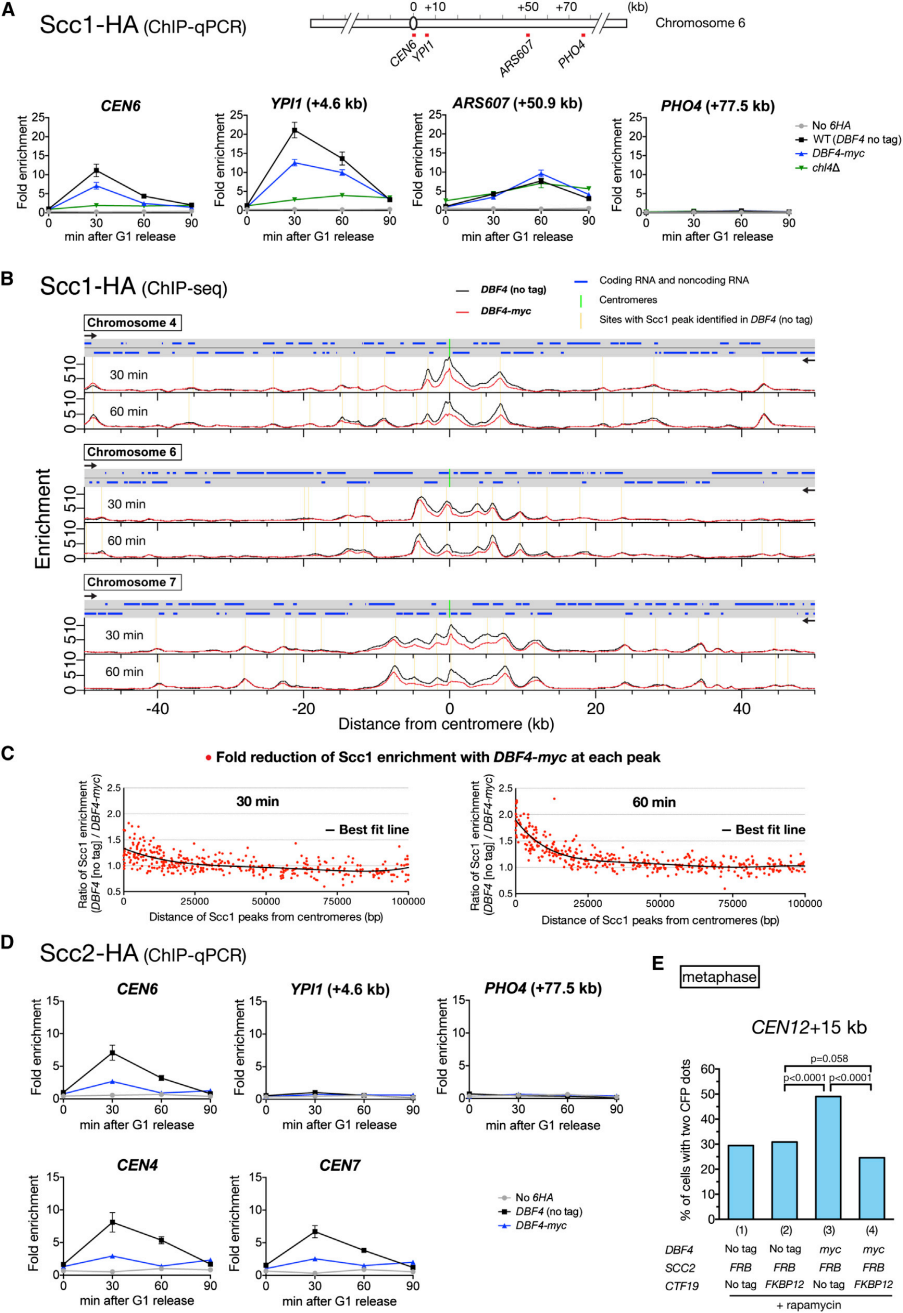
DDK at Kinetochores Facilitates Recruitment of Cohesin Loader and Enriches Cohesins at Pericentromeres (A) Cohesin Scc1 is reduced at a centromere (*CEN6*) and a pericentromere (*YPI1*), but not at a chromosome-arm site (*ARS607*) in *DBF4-myc* cells. *SCC1-HA* cells with WT (*DBF4* no tag) (T9882), *DBF4-myc* (T10007), and *chl4*Δ (T10561) were treated with α factor, released to fresh medium, aliquoted at indicated times, and processed for ChIP-qPCR (IP with an HA antibody). Cells lacking HA tags (T7107) were included as a control. Fold enrichment was as in Figure 3B (*CEN6* in WT at 0 min is set to 1). Error bars represent SD. (B) ChIP-seq analysis of Scc1 localization. T9882 and T10007 cells (see A) were treated as in (A) and analyzed by ChIP-seq (IP with an HA antibody). Scc1 peaks were identified using a peak-finding algorithm in cells with *DBF4* (no tag) at each time point. (C) Scc1 localization is specifically reduced up to 20 kb from centromeres. The ratio of Scc1 enrichment (*DBF4* [no tag]/*DBF4-myc*) at each peak identified in (B) is plotted against the distance from the centromere. The black line represents a regression curve. (D) The association of cohesin loader Scc2 with the centromere is reduced in *DBF4-myc* cells. *DBF4* (no tag) (T9883) and *DBF4-myc* (T10008) cells with *SCC2-HA* were analyzed as in (A). Error bars represent SD. (E) Artificial tethering of Scc2-FRB to Ctf19-FKBP12 strengthens pericentromeric cohesion when DDK is reduced at kinetochores. Cells with indicated alleles of *DBF4*, *SCC2*, and *CTF19*, as well as *tetOs* at +15 kb from *CEN12*, were treated and analyzed as in Figure 5D. n = 365–428 in each condition. See also Figure S6.

Robust cohesion at pericentromeres is achieved by enrichment of cohesin complexes in these regions (Tanaka, 2002). We therefore addressed whether the enrichment of cohesins at pericentromeres is affected when DDK is reduced at kinetochores by using ChIP-qPCR. In *DBF4-myc* cells, Scc1 accumulation was reduced at the centromere and at the pericentromere (4.6 kb from *CEN6*), but not at the chromosome-arm locus, 30 min after release from α factor arrest (Figure 6A), which corresponded to late G1–early S phase (Figure S6A). To evaluate Scc1 reduction more globally in *DBF4-myc* cells, we next used ChIP-seq (Figure 6B). At 30 and 60 min, the Scc1 reduction was greatest in the vicinity of the centromeres, became smaller when farther away from them, and was present up to 20 kb from them (Figures 6B and 6C). Note that Scc1 reduction with *DBF4-myc* may have been underestimated at 30 min with ChIP at some pericentromeres (Figure S6B). These results suggest that the kinetochore-associated DDK facilitates the cohesin enrichment at pericentromeres.

Nonetheless, both the reduction of Scc1 (Figure 6A) and sister chromatid separation (Figure S5) at pericentromeres remained milder in *DBF4-myc* cells than in *chl4*Δ cells. This may be due to residual DDK at kinetochores with *DBF4-myc*. Alternatively, the Ctf19 complex may enhance pericentromeric cohesion, partly independently of DDK.

The Scc2–Scc4 cohesin loader is enriched at centromeres, which enhances loading of cohesins at pericentromeres (Hu et al., 2011 and references therein). We next addressed whether the kinetochore-associated DDK promotes Scc2–Scc4 loading onto centromeres by using ChIP-qPCR. In *DBF4-myc* cells, the Scc2 loading at the centromere was reduced considerably, 30 min after release from α factor arrest (Figure 6D), which corresponded to late G1–early S phase (Figure S6C). Consistent with this, Scc2 accumulation near SPB was also reduced considerably in *DBF4-myc* cells in microscopy (Figure S6D). Thus, DDK at kinetochores promotes recruitment of cohesin loaders to centromeres.

Is the reduced level of cohesin loaders at centromeres, found in *DBF4-myc* cells, a major cause of reduced cohesins and weakened sister chromatid cohesion at pericentromeres? If so, it should be possible to restore pericentromeric cohesion by artificially tethering Scc2 to kinetochores in the presence of *DBF4-myc*. This was indeed the case. Whereas the addition of FRB to Scc2 itself somewhat weakened pericentromeric cohesion (Figure 6E [1]; compare with Figure 5B), *DBF4-myc* exacerbated this weakness (Figure 6E [3]). Notably, this extra weakness of cohesion due to *DBF4-myc* was abolished with Ctf19-FKBP12 (Figure 6E [4]), which tethered Scc2-FRB to kinetochores in the presence of rapamycin. Thus, the centromere recruitment of cohesin loaders by the kinetochore-associated DDK facilitates robust sister chromatid cohesion at pericentromeres. We next addressed whether the kinase activity of DDK is required for centromere recruitment of cohesin loaders. When the DDK kinase activity was inhibited using *cdc7-as3* in the presence of its inhibitor, Scc2 localization at centromeric regions was reduced (Figure S6E). Thus, the DDK kinase activity is required for this process.

### DDK at Kinetochores Independently Promotes Both Early S Phase Replication and Sister Chromatid Cohesion at Pericentromeres

Our findings suggest that the kinetochore-associated DDK has two functions: (1) facilitating Sld3–Sld7 loading onto pericentromeric replication origins and early S phase replication of centromeric regions, and (2) promoting robust pericentromeric cohesion through Scc2–Scc4 loading onto centromeres. Are the two functions interlinked or does DDK at kinetochores deliver the two functions independently?

**Figure 7:**
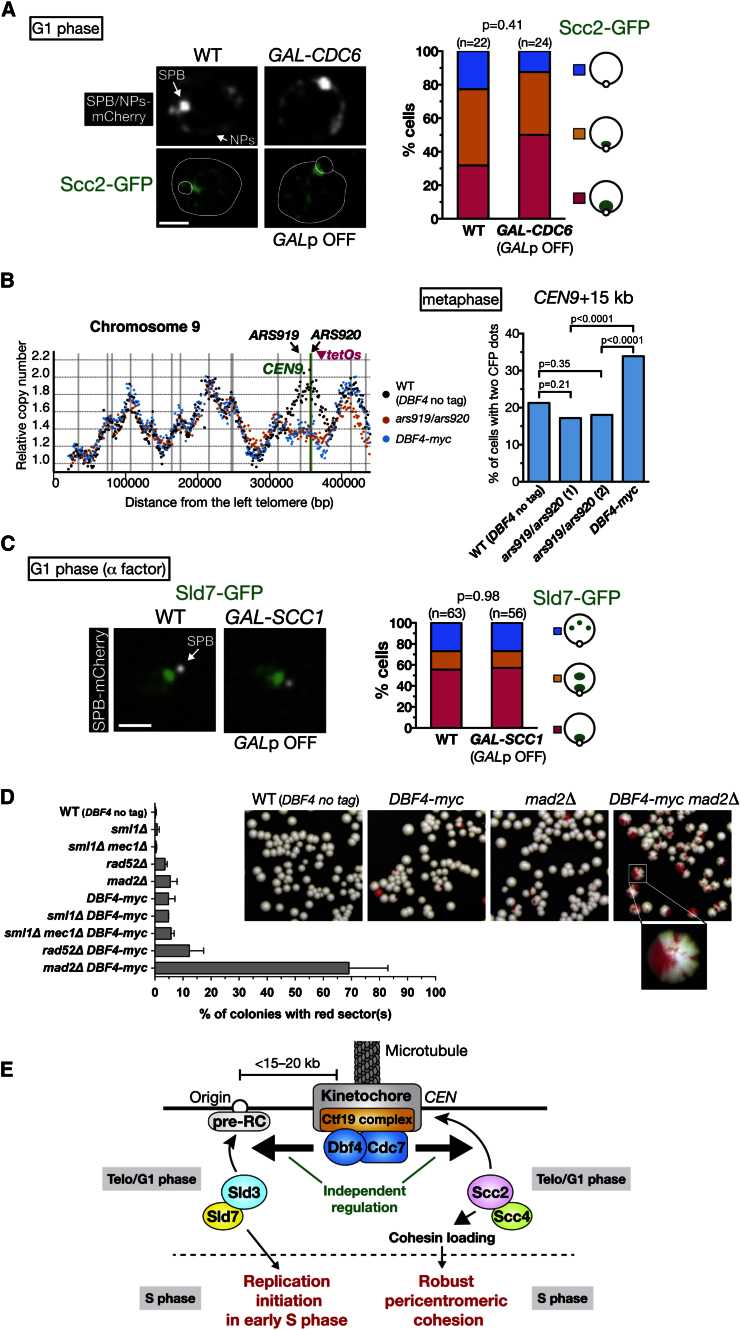
DDK at Kinetochores Independently Regulates Replication Timing and Cohesion at Pericentromeres (A) Scc2 accumulation at centromeric regions does not require the pre-RC in G1 phase. *CDC6*^*+*^ (WT, T10558) and *GAL-CDC6* (T10559) cells with *SCC2-GFP SPC42-mCherry NIC96-mCherry* were treated as in Figure 2Di. The scale bar represents 1 μm. (B) Inactivation of *CEN9*-proximal origins does not weaken cohesion at the *CEN9* pericentromere. WT (*DBF4* no tag, T10461, n = 282), *ars919*/*ars920* (clone 1, T10552, n = 314; clone 2, T10770, n = 316), and *DBF4-myc* (T10528, n = 295) cells with *tetOs* at +15 kb from *CEN9* were treated as in Figure 5A and analyzed at 160 min after release from α factor. Left: replication timing of chromosome 9. (C) Cohesin is not required for Sld7 accumulation at centromeric regions in G1 phase. *SCC1*^*+*^ (WT, T9110) and *GAL-SCC1* (T9107) cells with *SLD7-GFP SPC42-mCherry* were analyzed as in Figure 2Ci. (D) *DBF4-myc* cells show an increase in chromosome loss (p = 0.0006), which is further enhanced by deletion of *MAD2* (p < 0.0001). Indicated strains with the *CFIII* chromosome fragment were used for a chromosome loss assay. Loss of *CFIII* generated red sector in colonies. Error bars represent SD. (E) Summary of two functions of DDK recruited to kinetochores. See details in the text. See also Figure S7.

Our results suggest that robust pericentromeric cohesion is promoted by DDK at kinetochores, independently of replication timing at centromeric regions, for the following reasons. First, we found that DDK at kinetochores promoted robust cohesion similarly around *CEN12* and *CEN2*, whereas it advanced replication timing greatly at *CEN12* but only modestly at *CEN2* (Figures 5B and 5C). Second, the depletion of Cdc6, which is required for replication initiation and for Sld3–Sld7 loading onto pericentromeric origins (Figure 2Ci), did not change Scc2 loading at centromeric regions in G1 phase (Figures 7A and S7A). Third, the mutation of *ARS919* and *ARS920*, two replication origins supporting early S phase replication around *CEN9*, did not weaken sister chromatid cohesion at that region, in contrast to *DBF4-myc* (Figures 7B and S7B).

Moreover, Sld3–Sld7 accumulation and replication timing were not affected at centromeric regions when there was a change in the strength of cohesion or amount of cohesins, as shown by the following observations. First, we found that depletion of Scc1 did not change Sld7 accumulation at centromeric regions in G1 phase (Figure 7C) or initiation of replication-factory formation at these regions at S phase onset (Figure S7C). Second, although artificial tethering of Scc2 to kinetochores in a background of *DBF4-myc* restored robust cohesion at pericentromeres (Figure 6E), it failed to restore Sld7 accumulation at centromeric regions (Figure S7D). Third, after Scc2 was depleted, the Sld7 accumulation at centromeric regions was not changed (Figure S2A). Altogether, these results suggest that DDK at kinetochores regulates pericentromeric cohesion and replication timing, independently of each other.

Finally, we evaluated how DDK at kinetochores contributes to chromosome stability during cell proliferation. The chromosome loss rate was modestly enhanced with *DBF4-myc* (Figure 7D), wherein DDK was reduced at kinetochores (see Figure 4B). Intriguingly, the chromosome loss frequency increased synergistically when *DBF4-myc* was combined with *mad2*Δ (Figure 7D), wherein a spindle assembly checkpoint (SAC) was defective. In contrast, such an increase was negligible or remained additive when combined with *rad52*Δ and *mec1*Δ, wherein recombinational repair and the replication and DNA damage checkpoints were defective. Thus, reduction of DDK at kinetochores makes chromosome stability much more dependent on the SAC. Such chromosome instability with *DBF4-myc* was at least partly attributed to weakened pericentromeric cohesion (Eckert et al., 2007; Ng et al., 2009). Nonetheless, it is probable that a delay in the replication of centromeric regions also contributes to chromosome instability, as observed with mutations at pericentromeric origins *ARS919* and *ARS920* (Figure S7E).

## Discussion

### The Ctf19 Kinetochore Complex Recruits DDK to Kinetochores in Telophase–G1 Phase

We have found that, in telophase to early G1 phase, the Ctf19 complex promotes recruitment of DDK to kinetochores (Figure 7E). This DDK in turn promotes loading of Sld3–Sld7 onto pericentromeric origins (up to 15–20 kb from centromeres), which leads to early S phase replication of this region. Furthermore DDK at kinetochores also enhances Scc2–Scc4 recruitment to centromeres, which promotes cohesin loading at pericentromeres (up to 20 kb from centromeres) in late G1 phase. Both these functions of DDK require its kinase activity and are regulated independently of each other, suggesting that they rely on phosphorylation of different DDK substrates.

How does the Ctf19 complex recruit DDK to kinetochores? When kinetochores were purified from yeast cell extracts, Dbf4 was copurified together with multiple Ctf19-complex components (Akiyoshi et al., 2009), suggesting a physical interaction of DDK with kinetochores. Several lines of evidence suggest that Ctf19 complex plays a central role, among many kinetochore components, in recruiting DDK to kinetochores (Figures 3A, 4D, and 5D; Eckert et al., 2007). Components of the Ctf19 complex are evolutionarily conserved from yeast to humans (Schleiffer et al., 2012). It will be intriguing to investigate which component is directly involved in DDK recruitment to kinetochores and whether such function is conserved in evolution.

It has been thought that DDK has little, if any, role from telophase to early G1 phase given that Dbf4 is targeted for destruction by the anaphase-promoting complex/cyclosome (APC/C) with Cdh1 during this period (Ferreira et al., 2000; Weinreich and Stillman, 1999). From the onset of anaphase through to telophase, the amount of Dbf4 in the nucleus dramatically decreases (data not shown), but we found that a small amount of Dbf4 protein still localizes to kinetochores during telophase to G1 phase. We propose that the small fraction of Dbf4, associated with kinetochores, escapes APC/C-dependent degradation, which in turn promotes Sld3–Sld7 and Scc2–Scc4 recruitment in G1 phase. This is consistent with a previous report that, even if Dbf4 expression (and therefore DNA replication) is delayed until G2 phase, cohesins are still enriched at pericentromeres with normal kinetics in G1 phase (Beckouët et al., 2010). Thus, Dbf4 protein produced in the previous cell cycle is presumably sufficient for the DDK function required for cohesin enrichment (and probably for Sld3–Sld7 loading as suggested in Figure 2E) at pericentromeres. Nonetheless, DDK is still required continuously in G1 to govern replication timing and cohesion at pericentromeres (Figures 2E and S6E).

### DDK at Kinetochores Regulates Replication Timing and Sister Chromatid Cohesion at Pericentromeres

DDK at kinetochores facilitates Sld3–Sld7 loading on replication origins up to 15–20 kb from centromeres. What mechanism makes this possible considering that DDK at kinetochores seems to be spatially distant from such origins? This may be made possible by an intrachromatid loop called the C-loop, a distinct conformation formed by centromeric chromatin (Yeh et al., 2008). The C-loop may help to bring the pericentromeric regions into the vicinity of DDK at kinetochores. Interestingly, the C-loop extends up to 12–25 kb along both sides of a centromere, corresponding well with the range of the origins under regulation by DDK at kinetochores (Figures 4F and 4H). Alternatively, tethering kinetochores to a spindle pole by microtubules (Duan et al., 2010; Guacci et al., 1997) may enable DDK at kinetochores to act directly on pericentromeric origins on other chromosomes. Intriguingly, a small amount of Sld3–Sld7 is detected at centromeres (even if replication origins are not adjacent to them) in ChIP-seq, and this amount is reduced with *DBF4-myc* (Figure 4G). This is consistent with interaction between kinetochores and pericentromeric origins, which is envisaged in the above models.

When DDK was reduced at kinetochores, pericentromeric origins showed delays in initiating replication. What is the consequence of delayed pericentromere replication? It has been debated whether centromere replication in early S phase promotes kinetochore assembly (e.g., Koren et al., 2010). However, we did not find a reduction in the amount of kinetochore components (Mtw1 and Ndc80) in metaphase cells with *DBF4-myc* (data not shown). Nonetheless, kinetochores are disassembled and reassembled following centromere DNA replication in *S. cerevisiae* (Kitamura et al., 2007). Therefore, delayed centromere replication would delay kinetochore reassembly and shorten the time for establishing proper interaction with microtubules. Supporting this notion, the chromosome loss rate was enhanced when *ars919 ars920* mutants were combined with *mad2*Δ (Figure S7E), indicative of a defect in kinetochore-microtubule interaction.

Once DDK is recruited to kinetochores in telophase–early G1 phase, Sld3–Sld7 is rapidly recruited to the pericentromeric origins (Figure S1B). By contrast, Scc2–Scc4 accumulates at centromeres only in late G1 phase (Figure S6D). This is consistent with a recent finding that Scc2–Scc4 association at centromeres requires the cohesin Scc1, which accumulates in the nucleus during late G1 phase (Fernius et al., 2013).

Our results suggest that DDK governs both replication timing and cohesion at pericentromeres. Is there any advantage to regulating both these processes with the same kinase? For establishing robust cohesion at pericentromeres, cohesins must be loaded before replication of this region. On the other hand, if cohesins are not engaged in holding chromatids (i.e., not involved in cohesion), they are rapidly turned over (Gerlich et al., 2006; Lopez-Serra et al., 2013). Thus, in the natural environment where cell-cycle progression could be slow, cohesins might be lost at pericentromeres if cohesion is not established quickly. Thus, coupling the two processes, Scc2–Scc4 loading (thus cohesin loading) and early S phase replication (thus establishing cohesion earlier), may ensure robust sister chromatid cohesion at pericentromeres.

### The Roles of DDK in Replication Timing and Cohesion Might Be Conserved in Evolution but Could Be Used in Different Contexts

Are the roles of DDK in replication timing and sister chromatid cohesion conserved in evolution? In *S. pombe*, the heterochromatin protein Swi6 (ortholog of mammalian HP1) binds DDK, which advances replication timing of heterochromatin, including the pericentromere (Hayashi et al., 2009). It is also suggested that Swi6-bound DDK has a role in cohesin enrichment at heterochromatin (Bailis et al., 2003). Moreover, in *Xenopus* egg extracts, loading of Scc2 and cohesins to chromosomes depends on the pre-RC (Gillespie and Hirano, 2004; Takahashi et al., 2004) and DDK (Takahashi et al., 2008). These results suggest that the roles of DDK in advancing replication timing and in enhancing sister chromatid cohesion are conserved in evolution. However, *S. cerevisiae* and *C. albicans* lack canonical heterochromatin and a Swi6 ortholog; thus, early S phase replication of their centromeres (Koren et al., 2010; Raghuraman et al., 2001) cannot be explained by the heterochromatin-dependent mechanism. Moreover, in *Drosophila* cells, although their core centromeres are replicated in early S phase (Ahmad and Henikoff, 2001), the pericentromeric heterochromatin is replicated late; thus, HP1 does not seem to be involved in advancing replication timing, in contrast to *S. pombe*. Furthermore, it is unlikely that the pre-RC-dependent Scc2 loading in *Xenopus* egg extracts is present in *S. cerevisiae*, wherein Scc2 (Figure 7A) and cohesins (Uhlmann and Nasmyth, 1998) are still loaded on centromeres and chromosomes, respectively, after pre-RC formation is prevented by the depletion of Cdc6.

It seems, therefore, that the roles of DDK in replication timing and cohesion are conserved among different organisms but are used in different contexts, i.e., in heterochromatin-, pre-RC-, and kinetochore-dependent manners in *S. pombe*, *Xenopus* egg extracts, and *S. cerevisiae*, respectively. It is plausible that the kinetochore-dependent DDK recruitment is used not only in *S. cerevisiae* but also in other organisms, such as *C. albicans*, *Trypanosoma*, and *Drosophila*, wherein centromeres are replicated in early S phase (see references in Introduction). In mammalian cells, the replication timing of centromeres has been difficult to study because of their highly repetitive DNA sequences. However, at least within a mammalian neocentromere, the core region is replicated earlier than the surrounding region (Lo et al., 2001); thus, a kinetochore-dependent mechanism, similar to that in *S. cerevisiae*, may regulate replication timing of the core centromere.

## Experimental Procedures

Methods for yeast culture and FACS were described previously (Kitamura et al., 2006). Unless otherwise noted, cells were cultured at 25°C in YP medium containing glucose, and yeast genes were tagged at their C termini at their original gene loci with a one-step PCR method. *PCNA* was tagged at the N terminus and integrated at an auxotroph marker locus. In addition to the C-terminal tagging, *DBF4* was also tagged at the N terminus at the original gene locus. For activation and suppression of the *GAL1-10* promoter, cells were incubated in medium containing 2% galactose (plus 2% raffinose) or 2% glucose, respectively. The procedures for time-lapse microscopy (Kitamura et al., 2007), replication-timing analysis (Müller and Nieduszynski, 2012), and ChIP-seq (De Piccoli et al., 2012; Nakato et al., 2013) were described previously. See more details in the Supplemental Experimental Procedures.

## Accession Numbers

Replication-timing data have been deposited in the NCBI Gene Expression Omnibus under the accession numbers GSE42243 and GSE41982. ChIP-seq data have been deposited in the BioProject database under the accession number PRJNA193069. Both sets of data are available in graphic form at http://www.lifesci.dundee.ac.uk/other/files/DDK_MolCell_2013_suppl.pdf.

## Supplemental Information

Document S1. Supplemental Experimental Procedures and Figures S1–S7

## References

[bib1] Ahmad K., Henikoff S. (2001). Centromeres are specialized replication domains in heterochromatin. J. Cell Biol..

[bib2] Akiyoshi B., Nelson C.R., Ranish J.A., Biggins S. (2009). Quantitative proteomic analysis of purified yeast kinetochores identifies a PP1 regulatory subunit. Genes Dev..

[bib3] Aparicio O.M. (2013). Location, location, location: it’s all in the timing for replication origins. Genes Dev..

[bib4] Araki H. (2011). Initiation of chromosomal DNA replication in eukaryotic cells; contribution of yeast genetics to the elucidation. Genes Genet. Syst..

[bib5] Bailis J.M., Bernard P., Antonelli R., Allshire R.C., Forsburg S.L. (2003). Hsk1-Dfp1 is required for heterochromatin-mediated cohesion at centromeres. Nat. Cell Biol..

[bib6] Beckouët F., Hu B., Roig M.B., Sutani T., Komata M., Uluocak P., Katis V.L., Shirahige K., Nasmyth K. (2010). An Smc3 acetylation cycle is essential for establishment of sister chromatid cohesion. Mol. Cell.

[bib7] Chen J., Zheng X.F., Brown E.J., Schreiber S.L. (1995). Identification of an 11-kDa FKBP12-rapamycin-binding domain within the 289-kDa FKBP12-rapamycin-associated protein and characterization of a critical serine residue. Proc. Natl. Acad. Sci. USA.

[bib8] de Moura A.P., Retkute R., Hawkins M., Nieduszynski C.A. (2010). Mathematical modelling of whole chromosome replication. Nucleic Acids Res..

[bib9] De Piccoli G., Katou Y., Itoh T., Nakato R., Shirahige K., Labib K. (2012). Replisome stability at defective DNA replication forks is independent of S phase checkpoint kinases. Mol. Cell.

[bib10] Dimitrova D.S., Gilbert D.M. (1999). The spatial position and replication timing of chromosomal domains are both established in early G1 phase. Mol. Cell.

[bib11] Duan Z., Andronescu M., Schutz K., McIlwain S., Kim Y.J., Lee C., Shendure J., Fields S., Blau C.A., Noble W.S. (2010). A three-dimensional model of the yeast genome. Nature.

[bib12] Eckert C.A., Gravdahl D.J., Megee P.C. (2007). The enhancement of pericentromeric cohesin association by conserved kinetochore components promotes high-fidelity chromosome segregation and is sensitive to microtubule-based tension. Genes Dev..

[bib13] Fernius J., Marston A.L. (2009). Establishment of cohesion at the pericentromere by the Ctf19 kinetochore subcomplex and the replication fork-associated factor, Csm3. PLoS Genet..

[bib14] Fernius J., Nerusheva O.O., Galander S., Alves Fde.L., Rappsilber J., Marston A.L. (2013). Cohesin-dependent association of scc2/4 with the centromere initiates pericentromeric cohesion establishment. Curr. Biol..

[bib15] Ferreira M.F., Santocanale C., Drury L.S., Diffley J.F. (2000). Dbf4p, an essential S phase-promoting factor, is targeted for degradation by the anaphase-promoting complex. Mol. Cell. Biol..

[bib16] Gartenberg M. (2009). Heterochromatin and the cohesion of sister chromatids. Chromosome Res..

[bib17] Gerlich D., Koch B., Dupeux F., Peters J.M., Ellenberg J. (2006). Live-cell imaging reveals a stable cohesin-chromatin interaction after but not before DNA replication. Curr. Biol..

[bib18] Gillespie P.J., Hirano T. (2004). Scc2 couples replication licensing to sister chromatid cohesion in Xenopus egg extracts. Curr. Biol..

[bib19] Guacci V., Hogan E., Koshland D. (1997). Centromere position in budding yeast: evidence for anaphase A. Mol. Biol. Cell.

[bib20] Hayashi M.T., Takahashi T.S., Nakagawa T., Nakayama J., Masukata H. (2009). The heterochromatin protein Swi6/HP1 activates replication origins at the pericentromeric region and silent mating-type locus. Nat. Cell Biol..

[bib21] Heller R.C., Kang S., Lam W.M., Chen S., Chan C.S., Bell S.P. (2011). Eukaryotic origin-dependent DNA replication in vitro reveals sequential action of DDK and S-CDK kinases. Cell.

[bib22] Hill A., Bloom K. (1989). Acquisition and processing of a conditional dicentric chromosome in Saccharomyces cerevisiae. Mol. Cell. Biol..

[bib23] Hu B., Itoh T., Mishra A., Katoh Y., Chan K.L., Upcher W., Godlee C., Roig M.B., Shirahige K., Nasmyth K. (2011). ATP hydrolysis is required for relocating cohesin from sites occupied by its Scc2/4 loading complex. Curr. Biol..

[bib24] Katou Y., Kaneshiro K., Aburatani H., Shirahige K. (2006). Genomic approach for the understanding of dynamic aspect of chromosome behavior. Methods Enzymol..

[bib25] Kim S.M., Dubey D.D., Huberman J.A. (2003). Early-replicating heterochromatin. Genes Dev..

[bib26] Kitamura E., Blow J.J., Tanaka T.U. (2006). Live-cell imaging reveals replication of individual replicons in eukaryotic replication factories. Cell.

[bib27] Kitamura E., Tanaka K., Kitamura Y., Tanaka T.U. (2007). Kinetochore microtubule interaction during S phase in Saccharomyces cerevisiae. Genes Dev..

[bib28] Koren A., Tsai H.J., Tirosh I., Burrack L.S., Barkai N., Berman J. (2010). Epigenetically-inherited centromere and neocentromere DNA replicates earliest in S-phase. PLoS Genet..

[bib29] Labib K. (2010). How do Cdc7 and cyclin-dependent kinases trigger the initiation of chromosome replication in eukaryotic cells?. Genes Dev..

[bib30] Lo A.W., Craig J.M., Saffery R., Kalitsis P., Irvine D.V., Earle E., Magliano D.J., Choo K.H. (2001). A 330 kb CENP-A binding domain and altered replication timing at a human neocentromere. EMBO J..

[bib31] Lopez-Serra L., Lengronne A., Borges V., Kelly G., Uhlmann F. (2013). Budding yeast Wapl controls sister chromatid cohesion maintenance and chromosome condensation. Curr. Biol..

[bib32] Müller C.A., Nieduszynski C.A. (2012). Conservation of replication timing reveals global and local regulation of replication origin activity. Genome Res..

[bib33] Nakato R., Itoh T., Shirahige K. (2013). DROMPA: easy-to-handle peak calling and visualization software for the computational analysis and validation of ChIP-seq data. Genes Cells.

[bib34] Nasmyth K., Haering C.H. (2009). Cohesin: its roles and mechanisms. Annu. Rev. Genet..

[bib35] Natsume T., Tanaka T.U. (2010). Spatial regulation and organization of DNA replication within the nucleus. Chromosome Res..

[bib36] Ng T.M., Waples W.G., Lavoie B.D., Biggins S. (2009). Pericentromeric sister chromatid cohesion promotes kinetochore biorientation. Mol. Biol. Cell.

[bib37] Pohl T.J., Brewer B.J., Raghuraman M.K. (2012). Functional centromeres determine the activation time of pericentric origins of DNA replication in Saccharomyces cerevisiae. PLoS Genet..

[bib38] Raghuraman M.K., Brewer B.J., Fangman W.L. (1997). Cell cycle-dependent establishment of a late replication program. Science.

[bib39] Raghuraman M.K., Winzeler E.A., Collingwood D., Hunt S., Wodicka L., Conway A., Lockhart D.J., Davis R.W., Brewer B.J., Fangman W.L. (2001). Replication dynamics of the yeast genome. Science.

[bib40] Schleiffer A., Maier M., Litos G., Lampert F., Hornung P., Mechtler K., Westermann S. (2012). CENP-T proteins are conserved centromere receptors of the Ndc80 complex. Nat. Cell Biol..

[bib41] Takahashi T.S., Yiu P., Chou M.F., Gygi S., Walter J.C. (2004). Recruitment of Xenopus Scc2 and cohesin to chromatin requires the pre-replication complex. Nat. Cell Biol..

[bib42] Takahashi T.S., Basu A., Bermudez V., Hurwitz J., Walter J.C. (2008). Cdc7-Drf1 kinase links chromosome cohesion to the initiation of DNA replication in Xenopus egg extracts. Genes Dev..

[bib43] Tanaka T.U. (2002). Bi-orienting chromosomes on the mitotic spindle. Curr. Opin. Cell Biol..

[bib44] Tanaka T., Cosma M.P., Wirth K., Nasmyth K. (1999). Identification of cohesin association sites at centromeres and along chromosome arms. Cell.

[bib45] Tanaka S., Nakato R., Katou Y., Shirahige K., Araki H. (2011). Origin association of Sld3, Sld7, and Cdc45 proteins is a key step for determination of origin-firing timing. Curr. Biol..

[bib46] Tiengwe C., Marcello L., Farr H., Dickens N., Kelly S., Swiderski M., Vaughan D., Gull K., Barry J.D., Bell S.D., McCulloch R. (2012). Genome-wide analysis reveals extensive functional interaction between DNA replication initiation and transcription in the genome of Trypanosoma brucei. Cell Rep..

[bib47] Uhlmann F., Nasmyth K. (1998). Cohesion between sister chromatids must be established during DNA replication. Curr. Biol..

[bib48] Weber S.A., Gerton J.L., Polancic J.E., DeRisi J.L., Koshland D., Megee P.C. (2004). The kinetochore is an enhancer of pericentric cohesin binding. PLoS Biol..

[bib49] Weinreich M., Stillman B. (1999). Cdc7p-Dbf4p kinase binds to chromatin during S phase and is regulated by both the APC and the RAD53 checkpoint pathway. EMBO J..

[bib50] Yeh E., Haase J., Paliulis L.V., Joglekar A., Bond L., Bouck D., Salmon E.D., Bloom K.S. (2008). Pericentric chromatin is organized into an intramolecular loop in mitosis. Curr. Biol..

