# Supplementary material for: Kinetochores Coordinate Pericentromeric Cohesion and Early DNA Replication by Cdc7-Dbf4 Kinase Recruitment
Source: Mol Cell. 2013 Jun 6;50(5):661–74. doi: 10.1016/j.molcel.2013.05.011 (PMC3679449; doi:10.1016/j.molcel.2013.05.011)
Supplement: Document S1. Supplemental Experimental Procedures and Figures S1–S7 [file mmc1.pdf]

Molecular Cell, Volume *50*

Supplemental Information

Kinetochores Coordinate Pericentromeric Cohesion and Early DNA Replication by Cdc7-Dbf4 Kinase Recruitment

Toyoaki Natsume, Carolin A. Müller, Yuki Katou, Renata Retkute, Marek Gierliński, Hiroyuki Araki, J. Julian Blow, Katsuhiko Shirahige, Conrad A. Nieduszynski, and Tomoyuki U. Tanaka

Inventory of Supplemental Information

Figure S1 (related to Figure 2)	page 1
Figure S2 (related to Figure 3)	pages 2–3
Figure S3 (related to Figures 3 and 4)	page 4
Figure S4 (related to Figure 4)	pages 5–6
Figure S5 (related to Figure 5)	page 7
Figure S6 (related to Figure 6)	pages 8–9
Figure S7 (related to Figure 7)	pages 10–11
Supplemental Experimental Procedures	pages 12–21
Supplemental References	pages 22–25

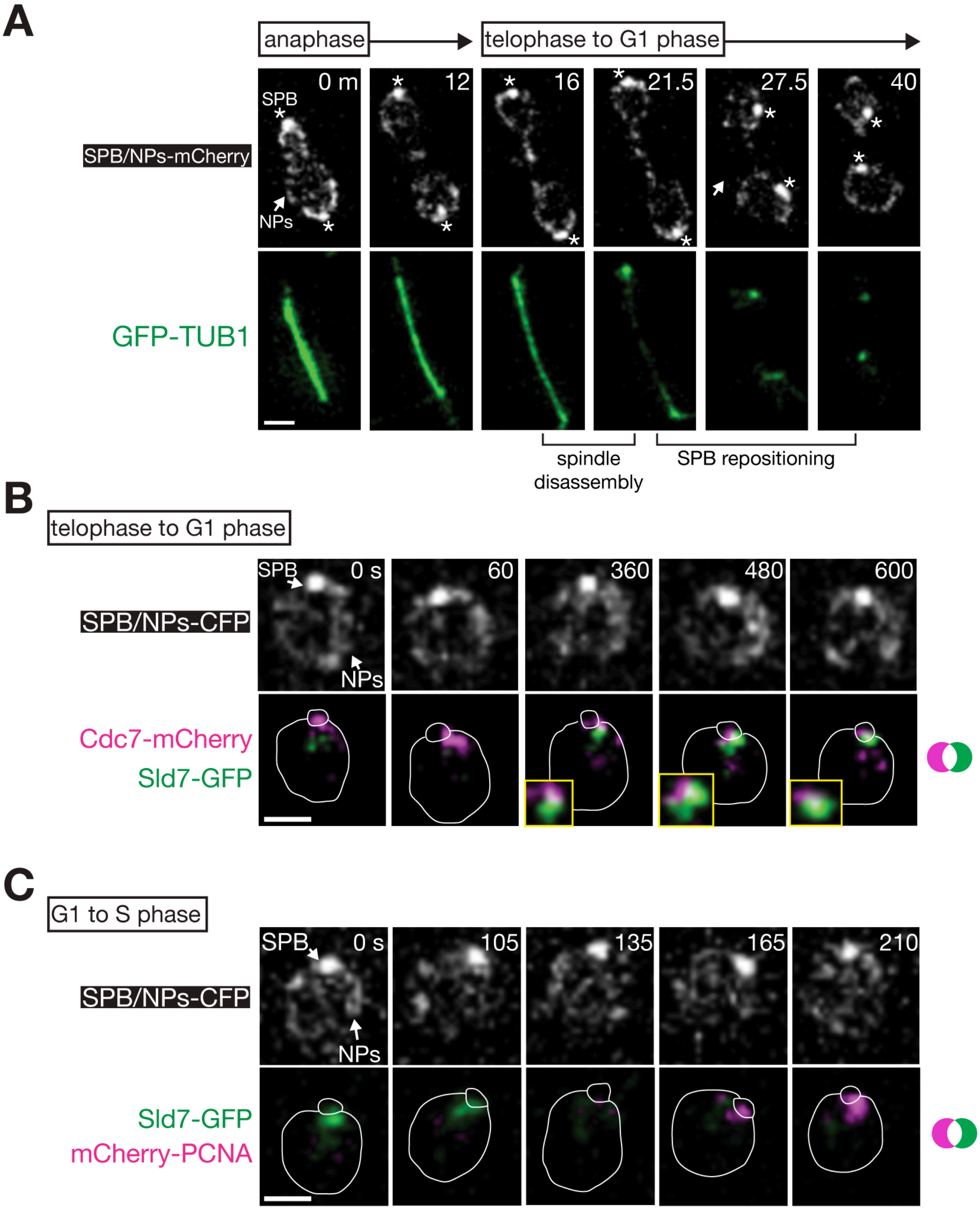

Figure S1. Supplemental Results about Subnuclear Localization of Replication Initiation Proteins during the Cell Cycle, Related to Figure 2

(A) Representative time-lapse images of a cell (T7637; *GFP-TUB1* [α-tubulin] and *SPC42-mCherry NIC96-mCherry*) progressing from anaphase to G1 phase in asynchronous culture. Size bar, 1 μm.

(B) Cdc7 and Sld7 signals appear in this order at centromeric regions from telophase to G1 phase. Representative time-lapse images of a cell (T9233) with *CDC7-mCherry SLD7-GFP SPC42-CFP NIC96-CFP* in asynchronous culture. Size bar, 1 μm.

(C) Disappearance of the Sld7 signals is followed by initial formation of replication factories on centromeric regions at the G1/S boundary. Representative time-lapse images of a cell (T8772) with *SLD7-GFP mCherry-PCNA SPC42-CFP NIC96-CFP* in asynchronous culture. Size bar, 1 μm.

A

Gene Name	Systematic Name	Function	Essential for Viability?	Mutation	Localization of Sld7 near SPB in telophase/G1 phase
BIR1	YJR089W	Survivin, Chromosome Passenger Complex	YES	*bir1-17*	+
BUB2	YMR055C	Mitotic exit network regulator	NO	*bub2Δ*	+
BUB3	YOR026W	Spindle assembly checkpoint	NO	*bub3Δ*	+
CAC1 (RLF2)	YPR018W	Chromatin assembly factor	NO	*cac1Δ*	+
CDC5	YMR001C	Polo-like Kinase	YES	*cdc5-aid**	+
CDC6	YJL194W	pre-replicative complex	YES	*GAL-CDC6*	-
CDC7	YDL017W	Catalytic subunit of Dbf4-dependent kinase	YES	*cdc7-4*	-
				cdc7-aid	-
				cdc7-as3	-
CHL4	YDR254W	Kinetochore (component of Ctf19 complex)	NO	*chl4Δ*	-
CNN1	YFR046C	Kinetochore	NO	*cnn1Δ*	+
CSM1	YMR048W	Monopolin complex	NO	*csm1Δ*	+
CTF3	YLR381W	Kinetochore (component of Ctf19 complex)	NO	*ctf3Δ*	-
CTF19	YPL018W	Kinetochore (component of Ctf19 complex)	NO	*ctf19Δ*	-
DBF4	YDR052C	Regulatory subunit of Dbf4-dependent kinase	YES	*dbf4-aid*	-
				dbf4-9myc	-
				dbf4-FRB	-
HIR1	YBL008W	Chromatin assembly factor	NO	*hir1Δ*	+
LRS4	YDR439W	Monopolin complex	NO	*lrs4Δ*	+
MAD2	YJL030W	Spindle assembly checkpoint	NO	*mad2Δ*	+
MAM1	YER106W	Monopolin complex	NO	*mam1Δ*	+
MCM21	YDR318W	Kinetochore (component of Ctf19 complex)	NO	*mcm21Δ*	-
MSA1	YOR066W	G1 transcription factor	NO	*msa1Δ*	+
NDC10 (CBF2)	YGR140W	Kinetochore (component of CBF3 complex)	YES	*ndc10-1*	-
ORC2	YBR060C	Origin recognition complex	YES	*GAL-orc2-1*	-
RIF1	YBR275C	Telomeric DNA binding	NO	*rif1Δ*	+/- (reduced)
RPD3	YNL330C	Histone deacetylase	NO	*rpd3Δ*	+
SCC1 (MCD1)	YDL003W	Cohesin SMC subunit	YES	*GAL-SCC1*	+
SCC2	YDR180W	Cohesin loader	YES	*scc2-aid*	+
SET2	YJL168C	Histone methyltransferase	NO	*set2Δ*	+
SGO1	YOR073W	Shugosin, Cohesin protector	NO	*sgo1Δ*	+
SIR1	YKR101W	Establishment of Silencing	NO	*sir1Δ*	+
SLK19	YOR195W	Kinetochore	NO	*slk19Δ*	+

* *aid*: auxin-inducible degron

B

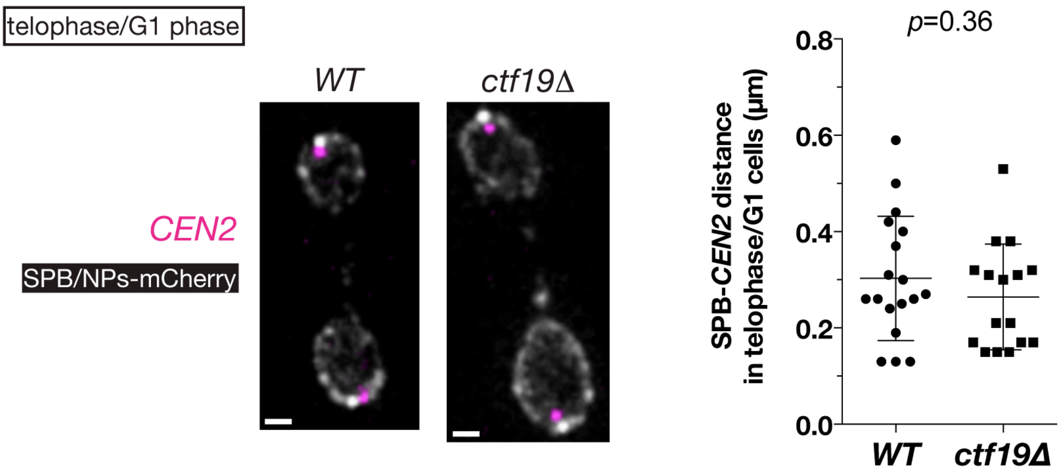

C

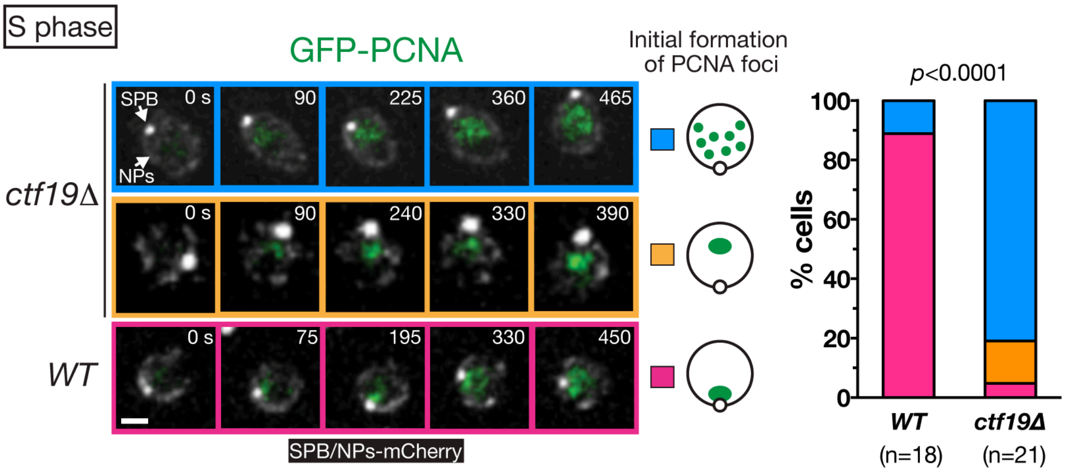

Figure S2. Supplemental Results about the Role of the Ctf19 Complex in Early Replication of Centromeric Regions, Related to Figure 3

(A) List of genes whose mutations were examined for Sld7 localization on centromeric regions in telophase to G1 phase. We visualized Sld7, rather than Sld3, because Sld7 showed higher signal intensity near the SPB in *wild-type* cells, although both showed a similar localization pattern. Genes marked in orange were required for the Sld7 localization near the SPB. These candidate regulators for DDK and Sld7 include the factors that are associated with centromeres/kinetochores and involved in DNA replication/sister chromatid cohesion. More specifically, we initially chose these candidate genes for screening for the following reasons. *BUB3, MAD2*; they may inhibit APC/C activity at centromeres in telophase/G1 phase. *CTF3*; it was identified in a screening of factors regulating initiation of DNA replication (Ma et al., 2010). *CDC5*, *BIR1, MSA1, RIF1, SLK19*; they associate with Cdc7 or Dbf4 in yeast two-hybrid or by co-immunopurification (Hardy and Pautz, 1996; Ho et al., 2002; Matos et al., 2008; Miller et al., 2009; unpublished results). *CAC1*, *HIR1*, *SIR1*; they facilitate formation of centromeric chromatin (Lopes da Rosa et al., 2011; Sharp et al., 2003). *CSM1*, *LRS4*, *MAM1* (monopolin complex); they are recruited to kinetochores in a DDK-dependent manner in meiosis, and Lrs4 is copurified with DDK (Lo et al., 2008; Matos et al., 2008). *RPD3*, *SET2*, *RIF1*; they regulate initiation time of replication origins (Knott et al., 2009; Lian et al., 2011; Pryde et al., 2009). *SCC1*, *SCC2*; they are enriched at centromeres and pericentromeres (Blat and Kleckner, 1999; Lengronne et al., 2004; Tanaka et al., 1999). Note that Ndc10 is an inner kinetochore component and, in *ndc10-1* mutant cells, centromeres detach from the spindle pole and the Ctf19 complex (and other kinetochore components) fails to localize at the centromere/kinetochore (De Wulf et al., 2003; Goh and Kilmartin, 1993).

(B) Centromere clustering near the SPB is not affected in *ctf19*Δ cells. *Wild-type* (*WT*, T9800) and *ctf19*Δ (T9801) cells with *CEN2-tetOs tetR-CFP SPC42-mCherry NIC96-mCherry* cells were observed by time-lapse imaging in asynchronous culture, and cells in telophase to G1 phase were selected. The mean distance between the SPB and *CEN2* at three consecutive time points was calculated just before SPB repositioning (Figure S1A), which followed spindle disassembly. The error bars indicate standard deviation. Size bar, 1 μm.

(C) Replication factories were initially formed near the SPB in fewer *ctf19*Δ cells. *Wild-type* (*WT*, T8375) and *ctf19*Δ (T9681) cells with *GFP-PCNA SPC42-mCherry NIC96-mCherry* were observed by time-lapse imaging in asynchronous culture. The patterns of initial replication factory formation were classified as near SPB (magenta), another site (orange), and multiple sites (blue). Size bar, 1 μm.

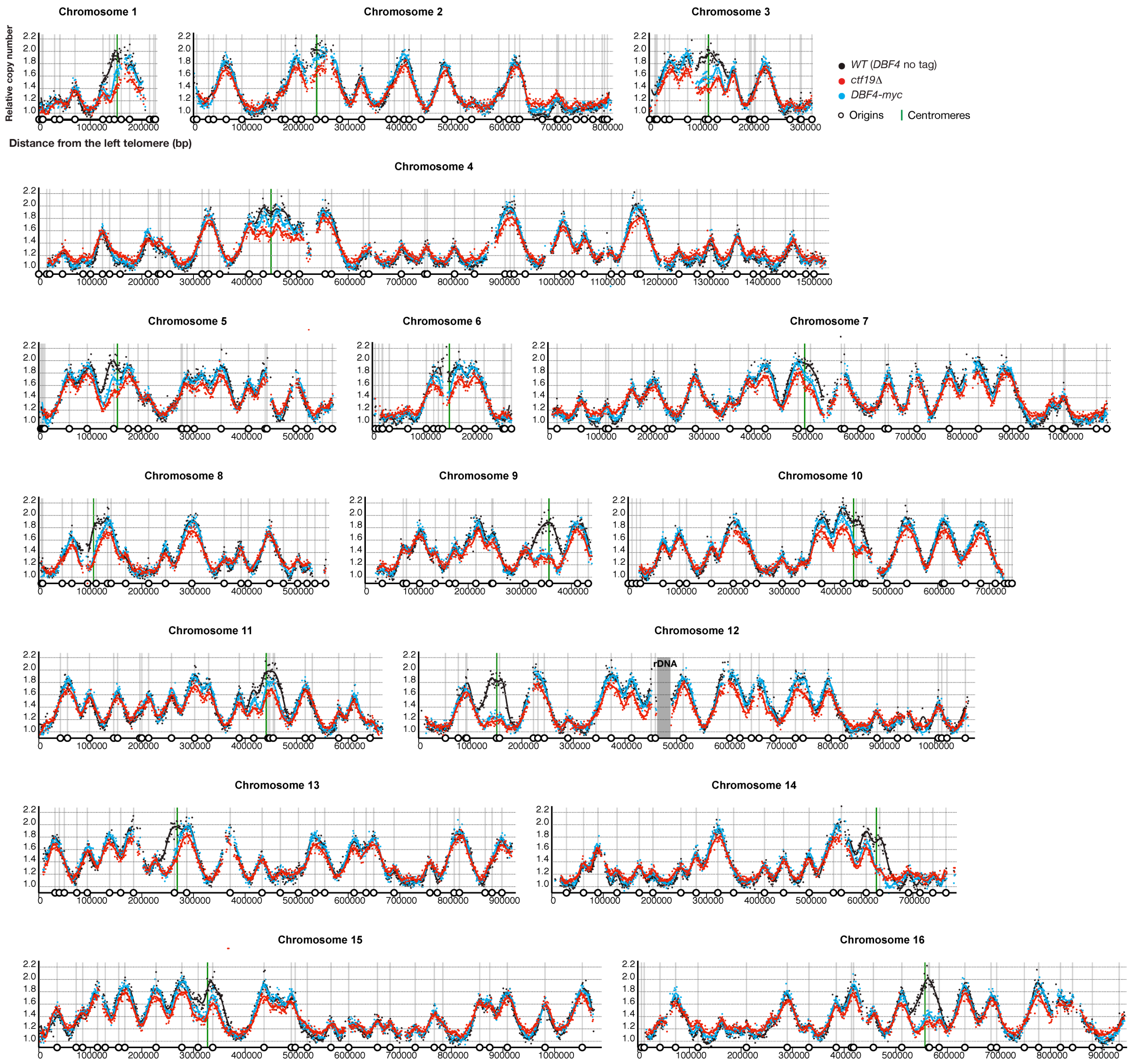

Figure S3. Whole-Genome Replication-Timing Profile of *ctf19*Δ and *DBF4-myc* Cells, Related to Figures 3 and 4

Replication timing profile for the whole genome of *wild type* (*WT* [*DBF4* no tag], T9475, black dots), *ctf19*Δ (T10117, red dots) and *DBF4-myc* (T9476, blue dots) homozygous diploid cells. Smoothed lines are added to both graphs

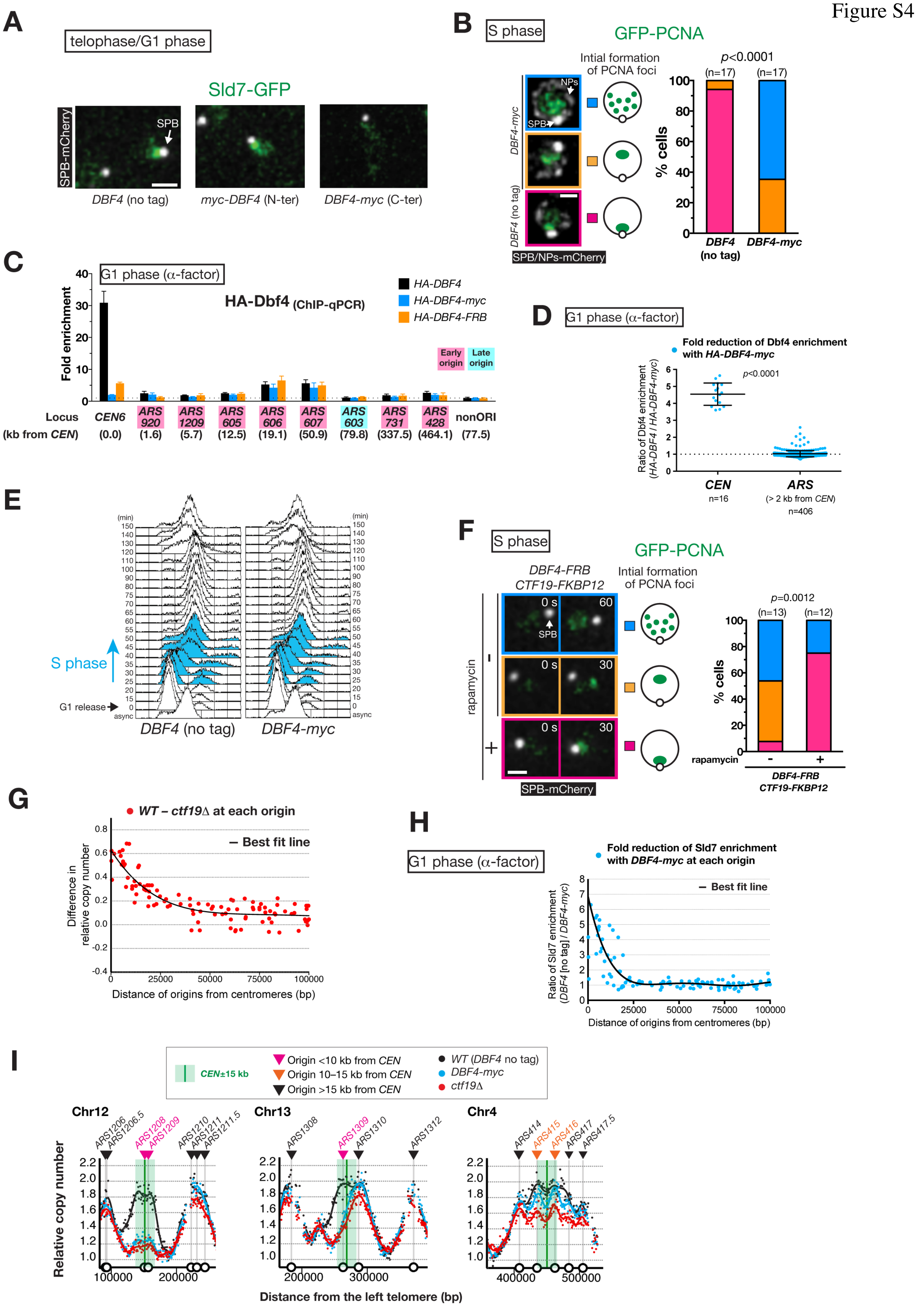
A
telophase/G1 phase
Sld7-GFP
SPB-mCherry
SPB
DBF4 (no tag)
myc-DBF4 (N-ter)
DBF4-myc (C-ter)
B
S phase
GFP-PCNA
Intial formation of PCNA foci
NPs
SPB
DBF4-myc
DBF4 (no tag)
SPB/NPs-mCherry
p<0.0001
(n=17) (n=17)
% cells
DBF4 (no tag)
DBF4-myc
C
G1 phase (α-factor)
HA-Dbf4 (ChIP-qPCR)
HA-DBF4
HA-DBF4-myc
HA-DBF4-FRB
Early origin
Late origin
Fold enrichment
Locus
(kb from CEN)
CEN6 (0.0)
ARS 920 (1.6)
ARS 1209 (5.7)
ARS 605 (12.5)
ARS 606 (19.1)
ARS 607 (50.9)
ARS 603 (79.8)
ARS 731 (337.5)
ARS 428 (464.1)
nonORI (77.5)
D
G1 phase (α-factor)
Fold reduction of Dbf4 enrichment with HA-DBF4-myc
p<0.0001
Ratio of Dbf4 enrichment (HA-DBF4 / HA-DBF4-myc)
CEN n=16
ARS (> 2 kb from CEN) n=406
E
(min)
S phase
G1 release
async
DBF4 (no tag)
DBF4-myc
F
S phase
GFP-PCNA
DBF4-FRB CTF19-FKBP12
Intial formation of PCNA foci
rapamycin
SPB
SPB-mCherry
p=0.0012
(n=13) (n=12)
% cells
rapamycin
DBF4-FRB CTF19-FKBP12
G
WT – ctf19Δ at each origin
Best fit line
Difference in relative copy number
Distance of origins from centromeres (bp)
H
G1 phase (α-factor)
Fold reduction of Sld7 enrichment with DBF4-myc at each origin
Best fit line
Ratio of Sld7 enrichment (DBF4 [no tag] / DBF4-myc)
Distance of origins from centromeres (bp)
I
CEN±15 kb
Origin <10 kb from CEN
Origin 10–15 kb from CEN
Origin >15 kb from CEN
WT (DBF4 no tag)
DBF4-myc
ctf19Δ
Chr12
Chr13
Chr4
ARS1206 ARS1206.5 ARS1208 ARS1209 ARS1210 ARS1211 ARS1211.5
ARS1308 ARS1309 ARS1310 ARS1312
ARS414 ARS415 ARS416 ARS417 ARS417.5
Relative copy number
Distance from the left telomere (bp)

Figure S4. Supplemental Results about the Role of DDK at Kinetochores in Early Replication of Centromeric Regions, Related to Figure 4

(A) Addition of tags to Dbf4 C-terminus reduces Sld7 accumulation at centromeric regions in telophase to G1 phase. *DBF4* (no tag) (T8613), *myc-DBF4* (N-terminally tagged, T9293), and *DBF4-myc* (C-terminally tagged, T9396) cells with *SLD7-GFP SPC42-mCherry* were observed by time-lapse imaging in asynchronous culture, and cells in telophase to G1 phase were selected. Size bar, 1 μm.

(B) Replication factories are initially formed near the SPB in fewer *DBF4-myc* cells. *DBF4* (no tag) (T8375) and *DBF4-myc* (T9395) cells with *GFP-PCNA SPC42-mCherry NIC96-mCherry* were observed by time-lapse imaging in asynchronous culture. The patterns of initial replication factory formation were classified as in Figure S2C. Size bar, 1 μm.

(C) The association of Dbf4 with replication origins in G1 phase. *HA-DBF4* (T9945), *HA-DBF4-myc* (T9979) and *HA-DBF4-FRB* (T10360) cells were treated and analyzed as in Figure 3C. The error bars indicate standard deviation.

(D) The association of Dbf4 with centromeres, but not with replication origins, is impaired when *DBF4* is tagged at its C-terminus. The ratio of Dbf4 enrichment between *HA-DBF4* and *HA-DBF4-myc* is plotted at each centromere (*CEN*) and replication origin (*ARS*) using ChIP-seq data. The error bars indicate standard deviation. Replication origins within 2 kb from centromeres were excluded from this analysis because the peak on these origins cannot be distinguished from the peak found on centromeres.

(E) FACS analysis of DNA content of *DBF4* (no tag) (T7107) and *DBF4-myc* (T9394) cells. The cells were treated with α factor and then released to fresh medium.

(F) Artificial tethering of Dbf4-FRB to Ctf19-FKBP restores the initial formation of replication factories near the SPB. Asynchronously growing cells with *GFP-PCNA DBF4-FRB CTF19-FKBP12 SPC42-mCherry* (T10216) were incubated in the presence and absence of 10 μM rapamycin and were observed by time-lapse imaging every 30 sec. 0 sec is the time point immediately before initial replication factory formation (appearance of globular GFP-PCNA signals). Upon S phase onset, the patterns of initial replication factory formation were classified as in Figure S2C. Size bar, 1 μm.

(G) Replication timing of origins in *wild-type* (*WT*) and *ctf19*Δ cells. Difference in replication timing (y-axis) between *wild-type* (*WT*) and *ctf19*Δ is plotted for origins against the distance from the centromere (x-axis). A regression curve is shown as a black line.

(H) Pericentromeric origins up to 15–20 kb from centromeres show reduction in their association with Sld7 in *DBF4-myc* cells. The ratio of Sld7 enrichment is plotted as in Figure 4H.

(I) The extent of a delay in replication of pericentromeres with *DBF4-myc* and *ctf19*Δ is explained by the positions of replication origins in the following three examples. At the *CEN12* pericentromere (left), two ARSs (*ARS1208* and *ARS1209*) are very close to *CEN12* and their replication initiation is severely delayed in *DBF4-myc* and *ctf19*Δ. In contrast, the next origins (*ARS1206.5* and *ARS1210*) are far outside of the pericentromere, resulting in a considerable delay in replication of this region. At the *CEN13* pericentromere (middle), *ARS1309* is very close to *CEN13* and its replication initiation is delayed in *DBF4-myc* and *ctf19*Δ cells. However, at the right hand side of *CEN13*, an early firing replication origin (*ARS1310*) is present just outside the ±15 kb centromeric region. Replication initiation at this origin (which occurs without a delay) reduces a delay in replication of the region at the right of *CEN13* in *DBF4-myc* and *ctf19*Δ cells. In comparison, at the left hand side of *CEN13,* the next early firing origin (*ARS1308*) is distant from *CEN13*. Thus, a delay in replication initiation at *ARS1309* leads to a delay in replication at the left of *CEN13,* without compensation, in *DBF4-myc* and *ctf19*Δ cells. At the *CEN4* pericentromere (right), two ARSs (*ARS415* and *ARS416*) are located within 10–15 kb from *CEN4* and their replication is modestly delayed in *DBF4-myc* and *ctf19*Δ cells. The next origins (*ARS414* and *ARS417*) are outside of ±15 kb region but they are relatively near *CEN4* in contrast to the case of the *CEN12* pericentromere. Such positioning of replication origins results in a modest delay in replication of this region.

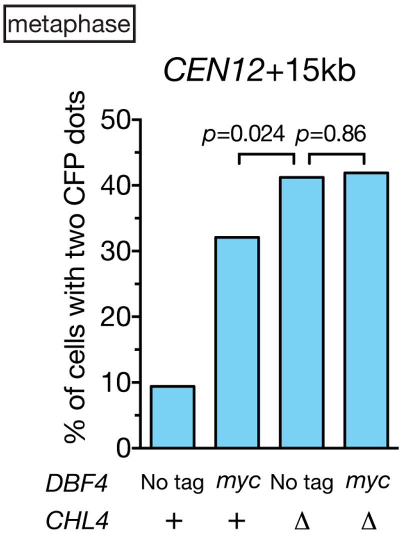

Figure S5. Supplemental Results about the Role of DDK at Kinetochores in Robust Pericentromeric Cohesion, Related to Figure 5

*chl4*Δ and *DBF4-myc chl4*Δ cells show similarly weakened pericentromeric cohesion. *DBF4* (no tag) *CHL4*$^{+}$ (T10141, n=395), *DBF4-myc* (T10142, n=327), *chl4*Δ (T10269, n=294), and *DBF4-myc chl4*Δ (T10270, n=267) cells with *tetOs* at +15 kb from *CEN12* were treated and analyzed as in Figure 5A. The weakness of pericentromeric cohesion in *chl4*Δ cells was not further enhanced by combination of *DBF4-myc* with *chl4*Δ. The result is consistent with the notion that the Ctf19 complex and DDK at kinetochores work in the same pathway to enhance pericentromeric cohesion.

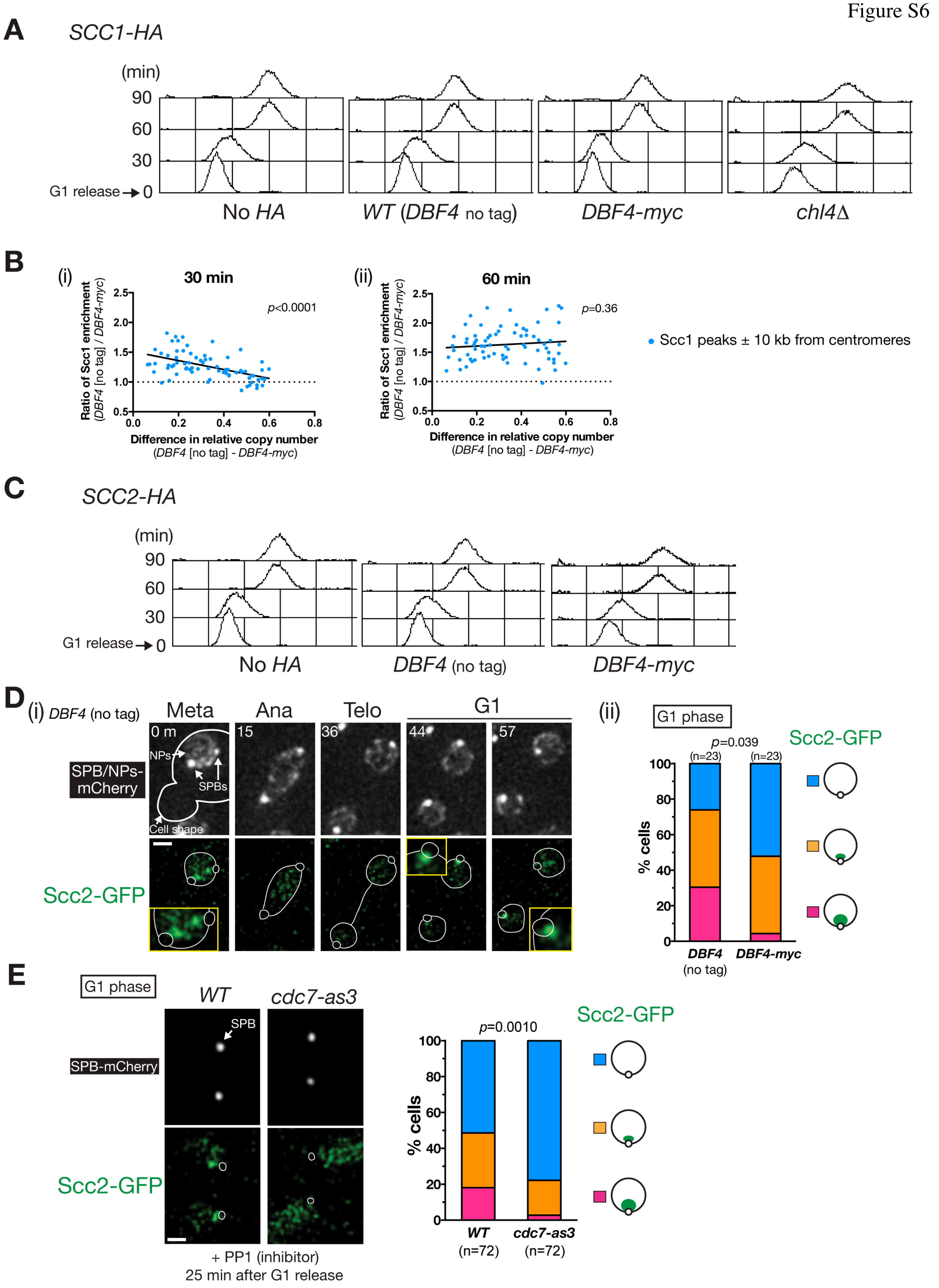
A
SCC1-HA
(min)
90
60
30
G1 release → 0
No HA
WT (DBF4 no tag)
DBF4-myc
chl4Δ
B
(i)
30 min
p<0.0001
Ratio of Scc1 enrichment
(DBF4 [no tag] / DBF4-myc)
Difference in relative copy number
(DBF4 [no tag] - DBF4-myc)
(ii)
60 min
p=0.36
Scc1 peaks ± 10 kb from centromeres
C
SCC2-HA
(min)
90
60
30
G1 release → 0
No HA
DBF4 (no tag)
DBF4-myc
D
(i) DBF4 (no tag)
Meta
Ana
Telo
G1
0 m
15
36
44
57
NPs
SPBs
Cell shape
SPB/NPs-mCherry
Scc2-GFP
(ii)
G1 phase
p=0.039
(n=23)
(n=23)
Scc2-GFP
% cells
DBF4
(no tag)
DBF4-myc
E
G1 phase
WT
cdc7-as3
SPB
SPB-mCherry
Scc2-GFP
+ PP1 (inhibitor)
25 min after G1 release
p=0.0010
% cells
WT
(n=72)
cdc7-as3
(n=72)
Scc2-GFP

Figure S6. Supplemental Results about the Role of DDK at Kinetochores in Cohesin Loading to Pericentromeres, Related to Figure 6

(A) FACS analysis of DNA content. Cells in Figure 6A were analyzed after release from α factor arrest.

(B) Negative correlation is found at pericentromeres of *DBF4-myc* cells between the delay in replication timing (x-axis) and the reduction in Scc1 enrichment (y-axis) at 30 min. The ratio of Scc1 enrichment (*DBF4* [no tag] / *DBF4-myc*), obtained in the ChIP-seq analysis at 30 min (i) and 60 min (ii), was plotted at each Scc1 peak within ±10 kb from centromeres, against the difference in replication timing at the same locus (copy number *DBF4* [no tag] - *DBF4-myc*; Figures 4C, S3). At 60 min, when most cells had completed S phase in both *DBF4* (no tag) and *DBF4-myc* (see [A]), there was no significant correlation between the Scc1 enrichment ratio and the difference in replication timing (ii). At 30 min, when the replication timing difference was small (< 0.2), the average Scc1 enrichment ratio (~1.5) was similar to that at 60 min. However, there was a tendency, at 30 min, for the Scc1 enrichment ratio to decrease (towards ~1.0) as the delay in replication timing with *DBF4-myc* increased (i). This tendency may be explained as follows: At 30 min after release from α factor, most cells were in early S phase (see [A]) when most centromeres would be replicated with *DBF4* (no tag) but some of them (e.g. *CEN9*, *CEN12*, and *CEN16*) would show a delay in replication with *DBF4-myc* (Figure S3). If pericentromeric DNA from the ChIP fraction did not increase proportionally to that in the WCE fraction immediately after DNA replication (e.g. the amount of associated Scc1 may not be doubled immediately after DNA amount being doubled due to replication, or the efficiency of Scc1 crosslinking to DNA may be reduced temporarily after replication), the replication delay with *DBF4-myc* would give a bias towards a higher value of ChIP/WCE (which gives Scc1 enrichment), relative to that with *DBF4* (no tag). In turn, this would give a bias towards a lower Scc1 enrichment ratio (*DBF4* [no tag] / *DBF4-myc*). Consistent with this, Cdc6 depletion (therefore replication inhibition) leads to a higher value of ChIP/WCE for Scc1, compared with *wild-type* cells (Figure S2 in Fernius et al., 2013). If the above explanation is correct, the reduction of Scc1 at pericentromeres with *DBF4-myc* was underestimated in ChIP at 30 min for a subgroup of pericentromeres where replication was delayed considerably.

(C) FACS analysis of DNA content. Cells in Figure 6D were analyzed after release from α factor arrest.

(D) Scc2 accumulation near the SPB in G1 phase becomes less frequent in *DBF4-myc* cells. (i) Time-lapse sequence of a representative cell with *SCC2-GFP SPC42-mCherry NIC96-mCherry* (T10339) from metaphase to G1 phase in asynchronous culture. Size bar, 1 µm. (ii) In *DBF4* (no tag) (T10342) and *DBF4-myc* (T10338) cells with *SCC2-GFP SPC42-mCherry*, patterns of Scc2 signals were classified in G1 phase as showing a strong focus (magenta), a weak focus (orange), and no focus near the SPB (blue). In control metaphase cells (untagged *DBF4*), Scc2 localized in the vicinity of separated SPBs, where bi-oriented sister centromeres should be clustered ([i] Meta), consistent with a previous report (Hu et al., 2011). These Scc2 foci disappeared at anaphase onset and reappeared in G1 phase ([i] Ana to G1). In the larger mother cell (top right), the Scc2 accumulation occurred earlier than in the smaller (bud-derived) daughter cell (bottom left) ([i] 44 min vs 57 min), which needed to spend longer growing before it reached START (late G1), suggesting that Scc2 accumulated near SPB in late G1 rather than in early G1. This is consistent with a recent finding that Scc2–Scc4 association at centromeres requires cohesin Scc1 that accumulates in the nucleus in late G1 (Fernius et al., 2013). The Scc2 foci in the proximity of the SPBs may indeed represent Scc2 localization at centromeres and we found that the Scc2 foci in G1 phase became considerably weaker in *DBF4-myc* cells (ii).

(E) Cdc7 kinase activity is required for Scc2 accumulation at centromeric regions in G1 phase. *Wild-type* (*WT*, T10574) and *cdc7-as3* (T10575) cells with *SCC2-GFP SPC42-4mCherry* were treated with α factor and released into medium with 20 µM PP1 (inhibitor). Images were acquired at 25 min after release from α factor (late G1 phase). Patterns of Scc2 signals were classified as in (D) ii. Size bar, 1 µm.

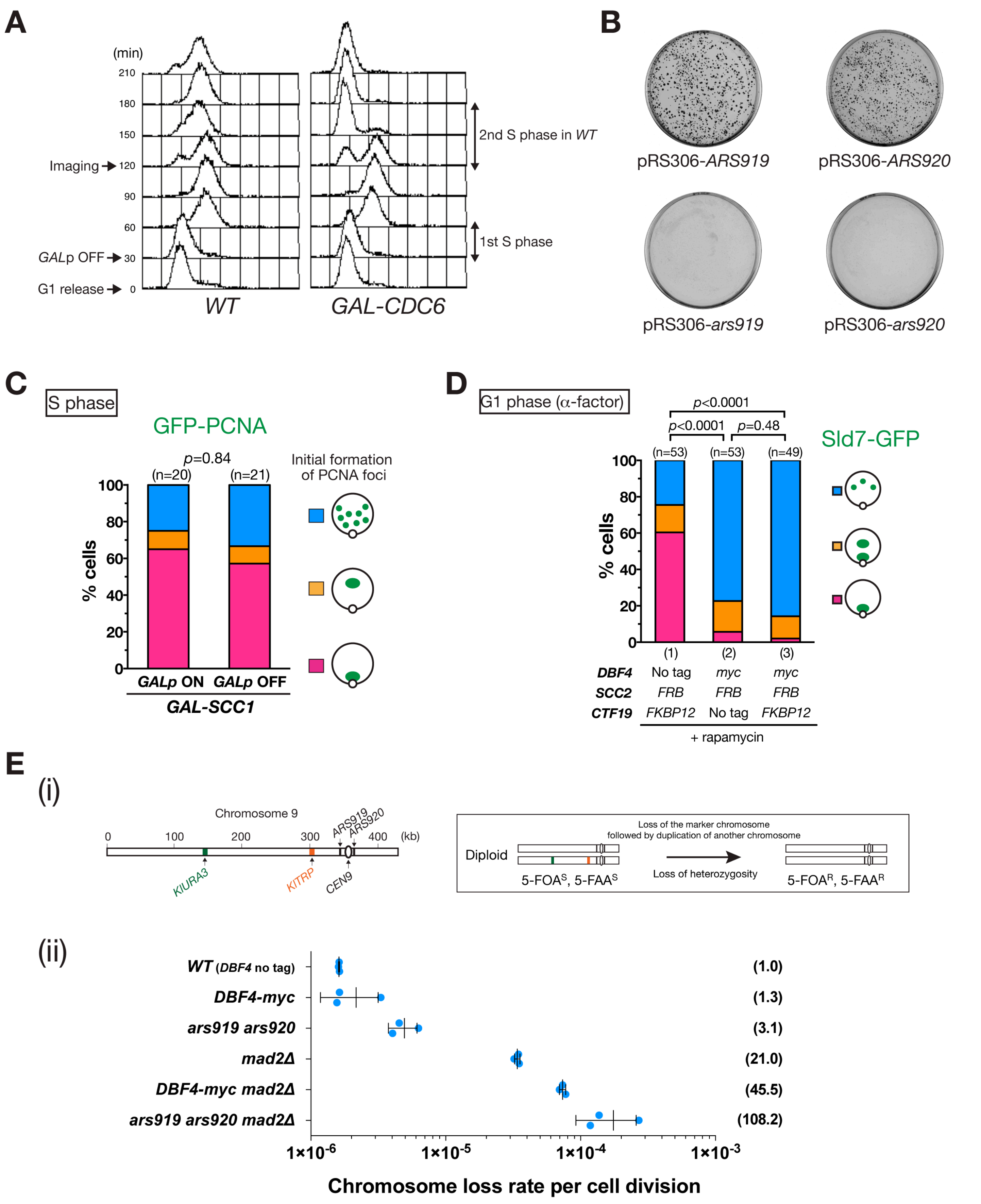
A
(min)
210
180
150
120
90
60
30
0
Imaging
GALp OFF
G1 release
2nd S phase in WT
1st S phase
WT
GAL-CDC6
B
pRS306-ARS919
pRS306-ARS920
pRS306-ars919
pRS306-ars920
C
S phase
GFP-PCNA
p=0.84
(n=20)
(n=21)
Initial formation of PCNA foci
% cells
GALp ON
GALp OFF
GAL-SCC1
D
G1 phase (α-factor)
p<0.0001
p<0.0001
p=0.48
(n=53)
(n=53)
(n=49)
Sld7-GFP
% cells
(1)
(2)
(3)
DBF4 No tag myc myc
SCC2 FRB FRB FRB
CTF19 FKBP12 No tag FKBP12
+ rapamycin
E
(i)
Chromosome 9
0
100
200
300
400
(kb)
ARS919
ARS920
KlURA3
KITRP
CEN9
Loss of the marker chromosome followed by duplication of another chromosome
Diploid
Loss of heterozygosity
5-FOAS, 5-FAAS
5-FOAR, 5-FAAR
(ii)
WT (DBF4 no tag) (1.0)
DBF4-myc (1.3)
ars919 ars920 (3.1)
mad2Δ (21.0)
DBF4-myc mad2Δ (45.5)
ars919 ars920 mad2Δ (108.2)
1×10-6
1×10-5
1×10-4
1×10-3
Chromosome loss rate per cell division

Figure S7. Supplemental Results about Independent Regulation of Replication Timing and Pericentromeric Cohesion by DDK at Kinetochores, Related to Figure 7

(A) FACS analysis of DNA content of *CDC6*$^{+}$ (*WT*, T10558) or *GAL-CDC6* (T10559) cells used in Figure 7A. Cells were treated with α factor in medium with raffinose and galactose (RG: *GAL*p ON). Cells were then released into fresh RG medium (G1 release). After 30 min (late G1/early S phase), they were transferred to medium with glucose (D: *GAL*p OFF) to start depletion of Cdc6.

(B) *ars919* and *ars920* mutations show loss of the ARS activity. The *ars919* and *ars920* mutations are 6 bp replacement within the core of the ARS consensus sequence (ACS) in *ARS919* and *ARS920* (Siow et al., 2012), respectively. Colony growth was analyzed on uracil drop-out medium, 4 days after *URA3* plasmids (pRS306) with *ARS919*, *ars919*, *ARS920*, or *ars920* had been used for transformation of *ura3* auxotroph cells.

(C) Cohesin is not required for initial formation of replication factories near the SPB. *GAL-SCC1* cells with *GFP-PCNA SPC42-mCherry* (T9109) were treated with α factor in medium with raffinose and galactose (RG). Cells were then released into RG medium without α factor. After 45 min, they were transferred to RG medium (*GAL*p ON) or D medium (*GAL*p OFF). After 75 min (after the majority of cells completed cytokinesis), they were observed every 20 sec with time-lapse imaging. Patterns of PCNA signals were classified as in Figure S2C.

(D) Artificial tethering of Scc2-FRB to Ctf19-FKBP in *DBF4-myc* cells failed to restore Sld7 localization at centromeric regions. *SLD7-GFP SPC42-mCherry NIC96-mCherry* cells with the indicated alleles of *DBF4*, *SCC2*, and *CTF19* ([1] T10584, [2] T10585, [3] T10587) were treated with α factor for 2.5 hrs in the presence of 10 μM rapamycin. Sld7 localization patterns were classified as exclusively near SPB (magenta), near SPB and other sites (orange), and no focus near SPB (blue).

(E) Loss rate of chromosome 9 in *ars919 ars920* and *DBF4-myc* cells in the presence and absence of spindle assembly checkpoint function. (i) An assay to evaluate the loss rate of chromosome 9. It was shown that loss of one chromosome 9 is followed by duplication of another chromosome 9, resulting in loss of heterozygosity (Reid et al., 2008). (ii) The delay in replication of centromeric regions causes chromosome instability. Chromosome loss rate per cell division was calculated from three independent experiments, using *wild-type* (*WT*; *DBF4* no tag) (T10649), *DBF4-myc* (T10917), *ars919 ars920* (T10650), *mad2*Δ (T10879), *DBF4-myc mad2*Δ (T10919) and *ars919 ars920 mad2*Δ (T10881) homozygous diploid cells with *KlURA3* and *KlTRP1* on one chromosome 9 as shown in (i). The error bars indicate standard deviation. Relative loss rate (*WT* is set to 1) is shown in parentheses (right). In contrast to the *CFIII* assay (Figure 7D), *DBF4-myc* did not show a significant increase in loss rate of chromosome 9 (p=0.39), suggesting that the effect on chromosome stability might be influenced by other factors such as a chromosome length (chromosome 9 is larger than *CFIII*). In contrast, *ars919 ars920* showed a slight increase in loss rate (p=0.0083) relative to *wild-type* although the delay in replication of the centromeric region on chromosome 9 was similar between *ars919 ars920* and *DBF4-myc* in the replication profile (Figure 7B). The difference between *ars919 ars920* and *DBF4-myc* may be explained as follows: Because both origins are completely inactive in *ars919 ars920*, in contrast to *DBF4-myc* in which they may still fire later in S phase (indeed small peaks were found at pericentromeric origins with *DBF4-myc*; Figure S3), a small population of *ars919 ars920* cells may experience an extreme delay in replication of the centromeric region if neighboring origins fail to fire, and these cells could contribute to an increase in the loss rate of chromosome 9. Thus, even if *DBF4-myc* could both weaken pericentromeric cohesion and delay replication of centromeric regions, its total chromosome loss rate could be still lower than *ars919 ars920*. Remarkably, both *DBF4-myc* and *ars919 ars920* synergistically increased the chromosome loss rate in the absence of spindle checkpoint function (*mad2*Δ). These results suggest that early replication of centromeric regions as well as robust sister chromatid cohesion (Eckert et al., 2007; Ng et al., 2009) is important for high-fidelity establishment of proper kinetochore–microtubule attachment.

SUPPLEMENTAL EXPERIMENTAL PROCEDURES

Yeast genetics and molecular biology

All yeast strains are in the W303 genetic background with wild-type *RAD5* and *BUD4* genes (T7107), which are mutated in the original W303 strain (Fan et al., 1996; Voth et al., 2005), unless otherwise stated. Methods for yeast culture, treatment with α-factor, and FACS DNA content analysis were as described previously (Amberg et al., 2005; Tanaka et al., 2007). Cells were cultured at 25 ºC in YP medium containing 2% glucose, unless otherwise noted. To activate and suppress the *GAL1-10* promoter (*PGAL*), cells were incubated in medium containing 2% galactose (plus 2% raffinose) or 2% glucose, respectively. To activate and suppress the *MET3* promoter (*PMET*), cells were incubated in methionine-drop-out media or synthetic complete media plus additional 2 mM methionine (both with appropriate carbon source), respectively.

Constructs of *TetR-3×CFP* (Bressan et al., 2004), *PGAL-CDC6* (Piatti et al., 1996), *PGAL-orc2-1* (Shimada and Gasser, 2007), *PMET-CDC20* (Uhlmann et al., 2000), *PGAL-SCC1* (Uhlmann and Nasmyth, 1998), *CDC28-4×GFP* (Maekawa et al., 2003), *GFP-TUB1* (Straight et al., 1997), *POL1-3×GFP* (Kitamura et al., 2006), *MTW1-3×CFP* (Maure et al., 2011), *NDC80-3×CFP*, and *NET1-4×mCherry* (Renshaw et al., 2010) were previously described. In yeast strains with *PGAL-CDC6*, *PGAL-orc2-1, PMET-CDC20* or *PGAL-SCC1*, the relevant gene was the only functional gene in the cells. Deletion mutants were created by a one-step PCR method using *KlURA3* (pUG72), *KlLEU2* (pUG73), *kanMX6* (pUG6), *KlTRP1* (pYM23), or *hphNT1* (pFA6a-hphNT1) cassettes as PCR templates (Guldener et al., 1996; Janke et al., 2004). Mutant alleles *cdc7-4* (Bousset and Diffley, 1998), *bir1-17* (Makrantoni and Stark, 2009), and *ndc10-1* (Goh and Kilmartin, 1993) were previously reported. To inhibit the kinase activity of Cdc7, cells with the *cdc7-as3* allele were treated with 20 μM of PP1 (4-amino-5-[4-methylphenyl]-7-[t-butyl]pyrazolo-d-3,4-pyrimidine, Merck), as described previously (Wan et al., 2006).

CDC7, *SLD3*, *SLD7*, *SLD2*, *CDC45,* and *CLB5* were tagged with three tandem copies of *GFP*; *CDC7*, *SPC42*, and *NIC96* with four tandem copies of *mCherry*; *SCC2* with a single copy of *GFP*; *SLD3*, *SLD7*, *SCC1*, and *SCC2* with *6×HA* at their C-termini at

their original gene loci by a one-step PCR method, using *3×GFP-kanMX6* (pSM1023; Maekawa et al., 2003), *4×mCherry-natMX6* (pT909), *GFP-CaURA3* (pKT209; Sheff and Thorn, 2004), and *6×HA-natNT2* (pYM17; Janke et al., 2004) cassettes as PCR templates, respectively. pT909 was constructed by multiplying the *mCherry* gene in pKS391 (Snaith et al., 2005). *DBF4* was tagged at its N-terminus at its original locus by a two-step "pop-in and pop-out" method (Struhl, 1983), using *6×HA* (pT1892), *9×myc* (pT1698), and *3×GFP* (pT1716). Briefly, a plasmid, which carries *URA3* marker and *DBF4* gene tagged at its N-terminus, was linearized within the *DBF4* sequence and integrated at the original *DBF4* locus (selected on uracil drop-out media), resulting in tandem *DBF4* copies there i.e. untagged and tagged ones. Spontaneous recombination between these copies, which leads to excision of the plasmid backbone including *URA3* and one copy of *DBF4,* was counter-selected on 5-FOA media. Depending on the location of the recombination, the *DBF4* gene tagged at its N-terminus remained at its original locus. Alternatively, *DBF4* was tagged at its C-terminus with *9×myc* at its original locus by a one-step PCR method, using *9×myc-HIS3MX6* (pYM19) or *9×myc-hphNT1* (pYM20) cassettes (Janke et al., 2004). When a N-terminally tagged *PCNA* was the sole source of PCNA, cell growth was severely retarded (Kitamura et al., 2006). Therefore, *GFP-PCNA* (pT1056, *TRP1* and pT1285, *HIS3*) and *mCherry-PCNA* (pT1079, *TRP1*), both of which were tagged at their N-termini, were integrated at auxotroph marker loci with the original *PCNA* locus intact. These strains showed normal growth.

CDC5, *CDC7*, *DBF4* and *SCC2* were tagged with an auxin-inducible degron (*aid*; Nishimura et al., 2009) at their C-termini at their original gene loci. Their protein degradation was induced within cells carrying rice (*Oryza sativa*) *TIR1* (*OsTIR1*, under the control of a constitutive *ADH1* promoter) in the presence of 2 mM auxin IAA (indole-3-acetic acid).

To mark *CEN2*+14kb, *CEN9*+15kb, and *CEN12*+15kb pericentromeric loci with fluorescence dots, a 400–500 bp genomic fragment (spanning each insertion site) was inserted into a plasmid containing both 11.1 kb 256×*tetOs* (Michaelis et al., 1997) and an auxotroph marker, which was subsequently cut within the genomic fragment and inserted into each genomic locus.

We thank J.F.X. Diffley, M. Foiani, S.M. Gasser, J.E. Haber, P. Hieter, N.M. Hollingsworth, M. Kanemaki, J.V. Kilmartin, E. Kitamura, U.K. Laemmli, J-F. Maure, A.W. Murray, K. Nasmyth, S. Piatti, M.J. Renshaw, K.E. Sawin, E. Schiebel, M.J.R. Stark, D.J. Stillman, R.Y. Tsien, F. Uhlmann, and EUROSCARF for reagents.

Artificial tethering of Dbf4 and Scc2

To artificially tether Dbf4 and Scc2 to kinetochores, *DBF4* was tagged with *FRB* using *FRB-HIS3MX6* (pFA6a-FRB-His3MX6); *SCC2* with *FRB* or *FRB-GFP* using *FRB-HIS3MX6* (pFA6a-FRB-His3MX6) or *FRB-GFP-kanMX6* (pFA6a-FRB-GFP-kanMX6) cassettes, respectively; *CTF19*, *MTW1*, and *MIF2* with *2×FKBP-TRP1* cassette (pFA6a-2×FKBP12-TRP1) at their C-termini at their original gene loci by a one-step PCR method (Haruki et al., 2008). For these experiments, yeast strains additionally carried *TOR1-1*, which conferred rapamycin resistance, and *fpr1*Δ mutations. Tethering of *FRB* and *FKBP12* fusion proteins was facilitated in the presence of 10 μM of rapamycin. Strains and the number of cells analyzed in these experiments are as follows: Figure 5D (1) T10400: n=353, (2) T10404: n=305, (3) T10403: n=315, (4) T10402: n=317; Figure 6E (1) T10463: n=394, (2) T10553: n=428, (3) T10465: n=365, (4) T10466: n=374.

Detaching centromeres from microtubules

Kinetochores are transiently disassembled and lose microtubule attachment during S phase (Kitamura et al., 2007). By inhibiting new kinetochore–microtubule attachment during S phase in the presence of nocodazole, centromeres can be efficiently detached from microtubules and kept away from SPBs (Figures 1C, 2B). To do this, cells were arrested with α factor and then released into S phase in the presence of 15 μg/ml nocodazole. To let cells enter the next cell cycle in the absence of kinetochore–microtubule attachment, two mitotic checkpoint pathways, spindle assembly checkpoint and spindle orientation checkpoint, were bypassed by deleting *MAD2* and *BUB2*, respectively (Alexandru et al., 1999). This allowed us to observe localization of Cdc7 and Sld7 (telophase/G1 phase) and replication factories (the

second S phase) with centromeres kept away from SPB.

Depleting Cdc6 and Orc2

Cdc6 was depleted using *GAL-CDC6* allele (Piatti et al., 1996) and the depletion was commenced before entry to telophase/G1 phase, as follows. First, cells were treated with α factor in medium with raffinose and galactose (RG: *GAL*p ON) for 3 hrs. Cells were then released into fresh RG medium. After 30 min (late G1/early S phase), they were transferred to medium with glucose (D: *GAL*p OFF) to start depletion of Cdc6. Cells were observed under the microscope either in telophase/G1 phase (Figure 2D i, 7A) or after arrested with α factor again (Figure 2C i).

Orc2 was depleted using *GAL-orc2-1* allele (Shimada and Gasser, 2007) and the depletion was commenced before entry to telophase/G1 phase, as follows. Simultaneous deletion of checkpoint genes, *RAD9*, *RAD24*, and *MAD2*, allowed cells to go through mitosis after Orc2 depletion (Shimada and Gasser, 2007). First, cells were treated with α factor in medium with RG (*GAL*p ON) for 2 hrs. Cells were then transferred to D media (*GAL*p OFF) in the presence of α factor to start depletion of Orc2. After 1 hr, cells were released into fresh D media. Cells were observed under the microscope either in telophase/G1 phase (Figure 2D ii) or after arrested with α factor again (Figure 2C ii).

Microscopy and image analysis

In all microscopy experiments, live yeast cells were observed without any fixation. The procedures for time-lapse fluorescent microscopy were described previously (Tanaka et al., 2010). Time-lapse images were collected at 25 ºC (ambient temperature). For image acquisition, we used a DeltaVision RT microscope (Applied Precision), UPlanSApo 100x objective lens (Olympus; NA 1.40), a CoolSnap HQ CCD camera (Photometrics), and SoftWoRx software (Applied Precision). CFP, GFP, and mCherry signals were discriminated using 89006 multi-band filter set (Chroma). We acquired 7–11 z sections (0.7 µm apart), which were subsequently deconvoluted and analyzed with Volocity software (PerkinElmer). Single z-stack images were

shown in figures when co-localization of two proteins was examined; otherwise the images projected to two dimensions were shown. For presenting an overlap between two fluorescent signals in a single image, we used green and magenta colours, which gave a white colour when colocalized. Statistical analyses were carried out using Prism software (GraphPad), by choosing Fisher's exact test (Figures 2D, 5B, 5C, 5D, 6E, 7B, S5), *chi*-square test (Figures 2C, 2E, 4A, 4D, 7A, 7C, S2C, S4B, S4F, S6D-ii, S6E, S7C, S7D), and *t*-test (Figure S2B). We thank the Light Microscopy Facility in University of Dundee for technical help.

Chromosome loss assay

Chromosome loss assay (Figure 7D) was performed using the *CFIII* chromosome fragment, as reported previously (Spencer et al., 1990). Cells with *ade2* mutation and the *CFIII* chromosome fragment (*CEN3.L. YPH278, URA3 SUP11*) were cultured in medium containing a low concentration of adenine (i.e. no additional adenine to YP media). *SUP11* on *CFIII* suppresses the *ade2* mutation that renders cells to accumulate red pigments. Cells maintaining *CFIII* form white colonies, while those that had lost *CFIII* form red colonies or red sectors within a white colony. Strains used in the experiment are as follows: *WT* (*DBF4* no tag) (K5041), *sml1*Δ (T9958), *sml1*Δ *mec1*Δ (T9980), *rad52*Δ (T9977), *mad2*Δ (T9865), *DBF4-myc* (T9814), *sml1*Δ *DBF4-myc* (T9959), *sml1*Δ *mec1*Δ *DBF4-myc* (T9981), *rad52*Δ *DBF4-myc* (T9978), *mad2*Δ *DBF4-myc* (T9866). The error bars indicate standard deviation obtained from two independent experiments. Statistical analyses were carried out using Prism software (GraphPad) by choosing *t*-test. Cells with *sml1*Δ *mec1*Δ (Labib and De Piccoli, 2011), *rad52*Δ (San Filippo et al., 2008), and *mad2*Δ (Musacchio and Salmon, 2007) mutations are defective in replication/DNA damage checkpoint, recombinational repair, and spindle assembly checkpoint, respectively. *MEC1* is essential for cell viability, but a simultaneous deletion of *SML1*, the inhibitor of ribonucleotide reductase, makes *mec1*Δ cells alive.

To examine the loss rate of chromosome 9 (Figure S7E), *KlURA3* and *KlTRP1* genes were integrated at *HIS5* and near *YIL028W* loci on one chromosome 9 of diploid cells, by a one-step PCR method using pUG72 and pYM23, respectively. Simultaneous loss

of *KlURA3* and *KlTRP1* genes was selected on media containing both 5-FOA and 5-FAA, which are toxic to Ura^+ and Trp^+ cells, respectively. Cultures originating from single colonies are diluted and plated on media with or without 5-FOA 5-FAA, and the fraction of 5-FOA^R 5-FAA^R cells in the cultures was measured by counting the number of colonies that emerged. The rate of chromosome loss per cell division was calculated according to the median method as previously described (Lea and Coulson, 1949). Statistical analyses were carried out using Prism software (GraphPad) by choosing *t*-test.

Introducing mutations at replication origins

A ~1 kb genomic fragment containing wild-type *ARS919* or *ARS920* was inserted into pRS306 plasmid (*URA3* marker, no ARS/CEN) to give pRS306-*ARS919* (pT2010) and pRS306-*ARS920* (pT2011). Using these constructs, a central 6 bp of ARS consensus sequence (ACS) in each ARS (Siow et al., 2012) was replaced with *Xho*I site (Nieduszynski et al., 2006) using site-directed mutagenesis, which gave pRS306-*ars919* (pT2017) and pRS306-*ars920* (pT2018), respectively. To evaluate the activity of these origins, 250 ng of each plasmid was used for transformation of a *ura3* auxotroph strain, and Ura+ transformants were selected on uracil-drop-out media. The *ars919* and *ars920* mutations were introduced at their original loci by a two-step "pop-in and pop-out" method (see above) using pRS306-*ars919* (pT2017) and pRS306-*ars920* (pT2018), respectively.

Replication timing profile

Replication timing profiles were obtained for the whole genome (every 1 kb) using homozygous diploid strains as described previously (Müller and Nieduszynski, 2012). Briefly, S phase and G2/M phase cells were fractionated from asynchronously growing cultures by cell sorting on the basis of DNA content. Then, using high throughput DNA sequencing, the number of DNA sequence reads in S phase cells was quantified every 1 kb, relative to that in G2/M phase cells. The earlier a sequence is replicated in S phase, the more reads of the sequence are obtained in the S phase sample. Thus the number of reads, represented as between 1.0–2.0, can be interpreted

as relative replication timing in S phase. Smoothed lines were generated using a Fourier transformation (see the above reference). The locations of replication origins were shown in the profiles, based on the DNA replication origin database, OriDB (Siow et al., 2012). To test the statistical significance of the difference in replication timing, we calculated the z-score; i.e. by how many standard deviations the difference at each data point (every 1 kb) is above or below the mean of the genome-wide difference plots. Based on the z-score, we calculated the probability (*p*-value) that the pairs of data points (from two strains) belonged to the same population. Genomic locations where the pairs of data points have *p*-value ≤0.001 and ≤0.005 were shown as black and grey bars, respectively, on the top of the replication timing profiles. The details of these statistical analyses will be reported elsewhere. Difference in replication time at origins (Figures 4E, 4F, S4G) was obtained based on the smoothed lines and plotted against the distance from the centromere. A polynomial curve fit (polynomial order of 5) was used to generate the regression curve.

Mathematical modeling

We used an analytical model for the spatial dynamics of DNA replication as described previously (Retkute et al., 2011, 2012). Briefly, each replication origin was described by parameters defining its position on the chromosome, its activation time (the time during S phase at which the origin activates) and its competence (the fraction of cells in which the origin is biochemically competent to fire – i.e. licensed). For each *DBF4* (no tag) and *DBF4-myc* strain, the parameters were estimated by minimizing the sum of the square of differences between model-calculated and experimental replication profiles. Origin firing probability distributions were compared in order to determine which origins showed a delay in activation time.

ChIP-qPCR

The procedures are based on the methods described previously (Tanaka et al., 1997) with some modifications. Yeast cells were treated with α factor for 2.5 hrs or released from the arrest (50 ml, 1.0–2.0 × 10^7 cell/ml). For crosslinking, cells were incubated with 1% formaldehyde for 20 min at 25 °C. Glycine was added to a final

concentration of 125 mM and the incubation continued for 10 min. Cells were harvested and washed three times with ice-cold TBS, and 1.25 × 10^8 cells were transferred to each 1.5 ml tube. For cell breakage, 300 μl of lysis buffer and glass beads were added to the tube (see the above reference). The cell lysate was sonicated either with Soniprep 150 (MSE, 20 sec × 12 times with the power level 7) or Bioruptor (Diagenode, 30 sec × 30 cycles with 30 sec intervals with high intensity), resulting in an average fragment size of 300 bp or 200 bp, respectively. The lysate was clarified by centrifugation at 13,000 g for 5 min (whole cell lysate, WCE). For immunoprecipitation, 60 μl of magnetic beads (Dynabeads Pan Mouse IgG, Invitrogen) were added following pre-incubatioin with an anti-HA monoclonal antibody (HA.11, Covance). Precipitates were washed on beads and processed as described in the above reference. The immunoprecipitated DNA and input DNA in WCE were quantified with quantitative PCR using Rotor-Gene 6000 and SYBR Green PCR kit (Qiagen), following the manufactures protocols (two-step PCR protocol, three independent PCR reactions for each sample). The ratio of immunoprecipitated DNA to total DNA in WCE was normalized as described in each figure legend and shown as fold enrichment. In Figure 3B and 6A, the error bars indicate standard deviation obtained from three independent PCR reactions.

ChIP-seq and data analyses

Chromatin immunoprecipitation was performed as previously described (Katou et al., 2003). High throughput sequencing and data processing were carried out as previously described (De Piccoli et al., 2012; Nakato et al., 2013). In order to minimize any bias in high throughput sequencing, the number of sequence reads from the ChIP fraction was divided by that of the corresponding WCE fraction at each site after calibration to give “enrichment” values of the examined protein.

To obtain the ratio of Dbf4 enrichment at each replication origin between *HA-DBF4* and *HA-DBF4-myc*, the mean of enrichment values was calculated between ± 1 kb from each replication origin (Siow et al., 2012), and the mean in *HA-DBF4* was divided by that of *HA-DBF4-myc* (Figure S4D). The ratio of Dbf4 enrichment at each centromere was similarly calculated between ±1 kb from each centromere (Figure

S4D). The ratio of Sld3 and Sld7 enrichment at each origin between *DBF4* (no tag) and *DBF4-myc* was also calculated similarly (Figures 4H, S4H).

We identified peaks of Scc1 association in ChIP-seq data using the following peak-finding algorithm: We start with a region R containing an entire chromosome. The data are position along the chromosome, p_i, and enrichment value, c_i, $i = 1, \ldots, n$.

1. Find the maximum enrichment value c_m at position p_m within region R.
2. If $c_m > c_{\lim}$, store the discovered peak (p_m, c_m), otherwise stop the algorithm; here $c_{\lim}$ is an arbitrary limit.
3. Find the left edge of the peak, p_l; follow the decreasing enrichment value for $j = m - 1, m - 2, \ldots$ until the decrease is reversed; in order to ignore little wiggles in data we compare c_j with a window of width w, i.e. we continued until such $j = l$ that $c_j < \min\{c_k : j - w \le k < j\}$.
4. Find the right edge of the peak, p_r, for $j = m + 1, m + 2, \ldots$ using the same method.
5. Exclude data points $(p_k, c_k), k = l, l + 1, \ldots, r$ from region R.
6. Repeat steps 1-5.

The routine is repeated for each chromosome. There are two arbitrary parameters in this algorithm, the minimum enrichment value, $c_{\lim}$, and the window size, w. By running the algorithm with several values of these parameters and visually inspecting the results, we found that $c_{\lim} = 1.5$ and $w = 50$ give reasonable results. Note that $w = 50$ corresponds to the window width of 0.5 kb in our sampling. Below $c_{\lim} = 1.5$ the algorithm picks up a lot of small wiggles which are presumably due to noise. Below $w = 50$ we find many "double peaks", i.e. components of what seems to be one peak. This selection of algorithm parameters is conservative and it certainly misses some weak and complex peaks. However, our aim is not to identify and characterize every peak in Scc1 data, but to find a representative selection of regions of increased Scc1 association and compare their enrichment values between the *DBF4* (no tag) and the *DBF4-myc*. By visually inspecting the detected peaks we confirm that the algorithm with these parameters is sufficient for our purpose.

To obtain the ratio of Scc1 enrichment at each Scc1 peak, the mean of enrichment values was calculated between ± 1 kb from each peak identified in *DBF4* (no tag), and the mean in *DBF4* (no tag) was divided by that of *DBF4-myc*.

Statistical analyses were carried out using Prism software (GraphPad) by choosing *t*-test (Figure S4D) and Pearson correlation coefficients (Figure S6B). A polynomial curve fit (polynomial order of 5) was used to generate the regression curve. In Figure 4H and S4H, origins within 2 kb from centromeres were excluded from the regression analysis because Sld3–Sld7 peaks on these origins cannot be distinguished from the peak found on centromeres (see text).

SUPPLEMENTAL REFERENCES

Alexandru, G., Zachariae, W., Schleiffer, A., and Nasmyth, K. (1999). Sister chromatid separation and chromosome re-duplication are regulated by different mechanisms in response to spindle damage. EMBO J *18*, 2707-2721.

Amberg, D.C., Burke, D.J., and Strathern, J.N. (2005). Methods in yeast genetics (CSHL Press).

Blat, Y., and Kleckner, N. (1999). Cohesins bind to preferential sites along yeast chromosome III, with differential regulation along arms versus the centric region. Cell *98*, 249-259.

Bousset, K., and Diffley, J.F. (1998). The Cdc7 protein kinase is required for origin firing during S phase. Genes Dev *12*, 480-490.

Bressan, D.A., Vazquez, J., and Haber, J.E. (2004). Mating type-dependent constraints on the mobility of the left arm of yeast chromosome III. J Cell Biol *164*, 361-371.

De Piccoli, G., Katou, Y., Itoh, T., Nakato, R., Shirahige, K., and Labib, K. (2012). Replisome stability at defective DNA replication forks is independent of S phase checkpoint kinases. Mol Cell *45*, 696-704.

De Wulf, P., McAinsh, A.D., and Sorger, P.K. (2003). Hierarchical assembly of the budding yeast kinetochore from multiple subcomplexes. Genes Dev *17*, 2902-2921.

Eckert, C.A., Gravdahl, D.J., and Megee, P.C. (2007). The enhancement of pericentromeric cohesin association by conserved kinetochore components promotes high-fidelity chromosome segregation and is sensitive to microtubule-based tension. Genes Dev *21*, 278-291.

Fan, H.Y., Cheng, K.K., and Klein, H.L. (1996). Mutations in the RNA polymerase II transcription machinery suppress the hyperrecombination mutant hpr1 delta of Saccharomyces cerevisiae. Genetics *142*, 749-759.

Fernius, J., Nerusheva, O.O., Galander, S., Alves Fde, L., Rappsilber, J., and Marston, A.L. (2013). Cohesin-dependent association of scc2/4 with the centromere initiates pericentromeric cohesion establishment. Curr Biol *23*, 599-606.

Goh, P.Y., and Kilmartin, J.V. (1993). NDC10: a gene involved in chromosome segregation in Saccharomyces cerevisiae. J Cell Biol *121*, 503-512.

Guldener, U., Heck, S., Fielder, T., Beinhauer, J., and Hegemann, J.H. (1996). A new efficient gene disruption cassette for repeated use in budding yeast. Nucleic Acids Res *24*, 2519-2524.

Hardy, C.F., and Pautz, A. (1996). A novel role for Cdc5p in DNA replication. Mol Cell Biol *16*, 6775-6782.

Haruki, H., Nishikawa, J., and Laemmli, U.K. (2008). The anchor-away technique: rapid, conditional establishment of yeast mutant phenotypes. Mol Cell *31*, 925-932.

Ho, Y., Gruhler, A., Heilbut, A., Bader, G.D., Moore, L., Adams, S.L., Millar, A., Taylor, P., Bennett, K., Boutilier, K.*, et al.* (2002). Systematic identification of protein complexes in Saccharomyces cerevisiae by mass spectrometry. Nature *415*, 180-183.

Hu, B., Itoh, T., Mishra, A., Katoh, Y., Chan, K.L., Upcher, W., Godlee, C., Roig, M.B., Shirahige, K., and Nasmyth, K. (2011). ATP hydrolysis is required for relocating cohesin from sites occupied by its Scc2/4 loading complex. Curr Biol *21*, 12-24.

Janke, C., Magiera, M.M., Rathfelder, N., Taxis, C., Reber, S., Maekawa, H.,

Moreno-Borchart, A., Doenges, G., Schwob, E., Schiebel, E., *et al*. (2004). A versatile toolbox for PCR-based tagging of yeast genes: new fluorescent proteins, more markers and promoter substitution cassettes. Yeast *21*, 947-962.

Katou, Y., Kanoh, Y., Bando, M., Noguchi, H., Tanaka, H., Ashikari, T., Sugimoto, K., and Shirahige, K. (2003). S-phase checkpoint proteins Tof1 and Mrc1 form a stable replication-pausing complex. Nature *424*, 1078-1083.

Kitamura, E., Blow, J.J., and Tanaka, T.U. (2006). Live-cell imaging reveals replication of individual replicons in eukaryotic replication factories. Cell *125*, 1297-1308.

Kitamura, E., Tanaka, K., Kitamura, Y., and Tanaka, T.U. (2007). Kinetochore microtubule interaction during S phase in Saccharomyces cerevisiae. Genes Dev *21*, 3319-3330.

Knott, S.R., Viggiani, C.J., Tavare, S., and Aparicio, O.M. (2009). Genome-wide replication profiles indicate an expansive role for Rpd3L in regulating replication initiation timing or efficiency, and reveal genomic loci of Rpd3 function in Saccharomyces cerevisiae. Genes Dev *23*, 1077-1090.

Labib, K., and De Piccoli, G. (2011). Surviving chromosome replication: the many roles of the S-phase checkpoint pathway. Philos Trans R Soc Lond B Biol Sci *366*, 3554-3561.

Lea, D.E., and Coulson, C.A. (1949). The distribution of the numbers of mutants in bacterial populations. J Genetics *49*, 264–285.

Lengronne, A., Katou, Y., Mori, S., Yokobayashi, S., Kelly, G.P., Itoh, T., Watanabe, Y., Shirahige, K., and Uhlmann, F. (2004). Cohesin relocation from sites of chromosomal loading to places of convergent transcription. Nature *430*, 573-578.

Lian, H.Y., Robertson, E.D., Hiraga, S., Alvino, G.M., Collingwood, D., McCune, H.J., Sridhar, A., Brewer, B.J., Raghuraman, M.K., and Donaldson, A.D. (2011). The effect of Ku on telomere replication time is mediated by telomere length but is independent of histone tail acetylation. Mol Biol Cell *22*, 1753-1765.

Lo, H.C., Wan, L., Rosebrock, A., Futcher, B., and Hollingsworth, N.M. (2008). Cdc7-Dbf4 regulates NDT80 transcription as well as reductional segregation during budding yeast meiosis. Mol Biol Cell *19*, 4956-4967.

Lopes da Rosa, J., Holik, J., Green, E.M., Rando, O.J., and Kaufman, P.D. (2011). Overlapping regulation of CenH3 localization and histone H3 turnover by CAF-1 and HIR proteins in Saccharomyces cerevisiae. Genetics *187*, 9-19.

Ma, L., Zhai, Y., Feng, D., Chan, T.C., Lu, Y., Fu, X., Wang, J., Chen, Y., Li, J., Xu, K., *et al*. (2010). Identification of novel factors involved in or regulating initiation of DNA replication by a genome-wide phenotypic screen in Saccharomyces cerevisiae. Cell Cycle *9*, 4399-4410.

Maekawa, H., Usui, T., Knop, M., and Schiebel, E. (2003). Yeast Cdk1 translocates to the plus end of cytoplasmic microtubules to regulate bud cortex interactions. EMBO J *22*, 438-449.

Makrantoni, V., and Stark, M.J. (2009). Efficient chromosome biorientation and the tension checkpoint in Saccharomyces cerevisiae both require Bir1. Mol Cell Biol *29*, 4552-4562.

Matos, J., Lipp, J.J., Bogdanova, A., Guillot, S., Okaz, E., Junqueira, M., Shevchenko, A., and Zachariae, W. (2008). Dbf4-dependent CDC7 kinase links DNA replication to the segregation of homologous chromosomes in meiosis I. Cell *135*, 662-678.

Maure, J.F., Komoto, S., Oku, Y., Mino, A., Pasqualato, S., Natsume, K., Clayton, L.,

Musacchio, A., and Tanaka, T.U. (2011). The Ndc80 loop region facilitates formation of kinetochore attachment to the dynamic microtubule plus end. Curr Biol *21*, 207-213.

Michaelis, C., Ciosk, R., and Nasmyth, K. (1997). Cohesins: chromosomal proteins that prevent premature separation of sister chromatids. Cell *91*, 35-45.

Miller, C.T., Gabrielse, C., Chen, Y.C., and Weinreich, M. (2009). Cdc7p-Dbf4p regulates mitotic exit by inhibiting Polo kinase. PLoS Genet *5*, e1000498.

Müller, C.A., and Nieduszynski, C.A. (2012). Conservation of replication timing reveals global and local regulation of replication origin activity. Genome Res *22*, 1953-1962.

Musacchio, A., and Salmon, E.D. (2007). The spindle-assembly checkpoint in space and time. Nat Rev Mol Cell Biol *8*, 379-393.

Nakato, R., Itoh, T., and Shirahige, K. (2013). DROMPA: easy-to-handle peak calling and visualization software for the computational analysis and validation of ChIP-seq data. Genes Cells, in press.

Ng, T.M., Waples, W.G., Lavoie, B.D., and Biggins, S. (2009). Pericentromeric sister chromatid cohesion promotes kinetochore biorientation. Mol Biol Cell *20*, 3818-3827.

Nieduszynski, C.A., Knox, Y., and Donaldson, A.D. (2006). Genome-wide identification of replication origins in yeast by comparative genomics. Genes Dev *20*, 1874-1879.

Nishimura, K., Fukagawa, T., Takisawa, H., Kakimoto, T., and Kanemaki, M. (2009). An auxin-based degron system for the rapid depletion of proteins in nonplant cells. Nat Methods *6*, 917-922.

Piatti, S., Bohm, T., Cocker, J.H., Diffley, J.F., and Nasmyth, K. (1996). Activation of S-phase-promoting CDKs in late G1 defines a "point of no return" after which Cdc6 synthesis cannot promote DNA replication in yeast. Genes Dev *10*, 1516-1531.

Pryde, F., Jain, D., Kerr, A., Curley, R., Mariotti, F.R., and Vogelauer, M. (2009). H3 k36 methylation helps determine the timing of cdc45 association with replication origins. PLoS One *4*, e5882.

Reid, R.J., Sunjevaric, I., Voth, W.P., Ciccone, S., Du, W., Olsen, A.E., Stillman, D.J., and Rothstein, R. (2008). Chromosome-scale genetic mapping using a set of 16 conditionally stable Saccharomyces cerevisiae chromosomes. Genetics *180*, 1799-1808.

Renshaw, M.J., Ward, J.J., Kanemaki, M., Natsume, K., Nedelec, F.J., and Tanaka, T.U. (2010). Condensins promote chromosome recoiling during early anaphase to complete sister chromatid separation. Dev Cell *19*, 232-244.

Retkute, R., Nieduszynski, C.A., and de Moura, A. (2011). Dynamics of DNA replication in yeast. Phys Rev Lett *107*, 068103.

Retkute, R., Nieduszynski, C.A., and de Moura, A. (2012). Mathematical modeling of genome replication. Phys Rev E Stat Nonlin Soft Matter Phys *86*, 031916.

San Filippo, J., Sung, P., and Klein, H. (2008). Mechanism of eukaryotic homologous recombination. Annu Rev Biochem *77*, 229-257.

Sharp, J.A., Krawitz, D.C., Gardner, K.A., Fox, C.A., and Kaufman, P.D. (2003). The budding yeast silencing protein Sir1 is a functional component of centromeric chromatin. Genes Dev *17*, 2356-2361.

Sheff, M.A., and Thorn, K.S. (2004). Optimized cassettes for fluorescent protein tagging in Saccharomyces cerevisiae. Yeast *21*, 661-670.

Shimada, K., and Gasser, S.M. (2007). The origin recognition complex functions in

sister-chromatid cohesion in Saccharomyces cerevisiae. Cell *128*, 85-99.

Siow, C.C., Nieduszynska, S.R., Müller, C.A., and Nieduszynski, C.A. (2012). OriDB, the DNA replication origin database updated and extended. Nucleic Acids Res *40*, D682-686.

Snaith, H.A., Samejima, I., and Sawin, K.E. (2005). Multistep and multimode cortical anchoring of tea1p at cell tips in fission yeast. EMBO J *24*, 3690-3699.

Spencer, F., Gerring, S.L., Connelly, C., and Hieter, P. (1990). Mitotic chromosome transmission fidelity mutants in Saccharomyces cerevisiae. Genetics *124*, 237-249.

Straight, A.F., Marshall, W.F., Sedat, J.W., and Murray, A.W. (1997). Mitosis in living budding yeast: anaphase A but no metaphase plate. Science *277*, 574-578.

Struhl, K. (1983). The new yeast genetics. Nature *305*, 391-397.

Tanaka, K., Kitamura, E., Kitamura, Y., and Tanaka, T.U. (2007). Molecular mechanisms of microtubule-dependent kinetochore transport toward spindle poles. J Cell Biol *178*, 269-281.

Tanaka, K., Kitamura, E., and Tanaka, T.U. (2010). Live-cell analysis of kinetochore-microtubule interaction in budding yeast. Methods *51*, 206-213.

Tanaka, T., Cosma, M.P., Wirth, K., and Nasmyth, K. (1999). Identification of cohesin association sites at centromeres and along chromosome arms. Cell *98*, 847-858.

Tanaka, T., Knapp, D., and Nasmyth, K. (1997). Loading of an Mcm protein onto DNA replication origins is regulated by Cdc6p and CDKs. Cell *90*, 649-660.

Uhlmann, F., and Nasmyth, K. (1998). Cohesion between sister chromatids must be established during DNA replication. Curr Biol *8*, 1095-1101.

Uhlmann, F., Wernic, D., Poupart, M.A., Koonin, E.V., and Nasmyth, K. (2000). Cleavage of cohesin by the CD clan protease separin triggers anaphase in yeast. Cell *103*, 375-386.

Voth, W.P., Olsen, A.E., Sbia, M., Freedman, K.H., and Stillman, D.J. (2005). ACE2, CBK1, and BUD4 in budding and cell separation. Eukaryot Cell *4*, 1018-1028.

Wan, L., Zhang, C., Shokat, K.M., and Hollingsworth, N.M. (2006). Chemical inactivation of cdc7 kinase in budding yeast results in a reversible arrest that allows efficient cell synchronization prior to meiotic recombination. Genetics *174*, 1767-1774.
